# Transcriptomic and metabolomic changes associated with the induction and initiation of juice sacs in citrus fruit

**DOI:** 10.1007/s00425-026-05008-9

**Published:** 2026-05-05

**Authors:** Siwar Assili, Dor Haim, Adi Doron-Faigenboim, Ido Nir, Avi Sadka

**Affiliations:** 1 Department of Fruit Tree Sciences, The Institute of Plant Sciences, Agricultural Research Organization, The Volcani Center, Rishon LeZion, Israel; 2 The Robert H. Smith Institute of Plant Sciences and Genetics in Agriculture, The Robert H. Smith Faculty of Agriculture, Food and Environment, The Hebrew University of Jerusalem, Rehovot, Israel; 3 Department of Vegetables Field Crops Research, The Institute of Plant Sciences, Agricultural Research Organization, The Volcani Center, Rishon LeZion, Israel

**Keywords:** Citron, Transcriptome, Juice sac, Citrus fruit, Metabolites, Endocarp differentiation

## Abstract

**Main conclusion:**

Transcriptomic and metabolic comparisons reveal putative regulatory and metabolic differences underlying juice sac initiation in citrus fruit.

**Abstract:**

The edible portion of citrus fruits consists of juice sacs—specialized structures unique among fruits—that develop shortly after anthesis from the endocarp, originating from the innermost layers of the albedo. While their physiological and biochemical properties are well studied, the regulatory mechanisms controlling juice sac initiation remain poorly understood. In this study, we compared two cultivars of citron (*Citrus medica* L.)—the *Calabria* citron, which develops juice sacs normally, and the *Yemenite* citron, which does not—across four developmental stages: closed flowers, flowers at anthesis, and fruitlets at one and two weeks post-anthesis. We performed a comparative transcriptomic analysis of endocarp cells, followed by Weighted Gene Co-Expression Network Analysis (WGCNA) and a metabolomic analysis of whole ovaries and fruitlets. As expected, the *Calabria* endocarp exhibited higher expression of genes associated with cell wall formation, DNA replication, and cell proliferation, particularly two weeks post-anthesis. In contrast, stress-related genes were more abundant in the *Yemenite* endocarp. *Calabria* ovaries and fruitlets showed an increase in amino acids, whereas those of the *Yemenite* citron exhibited induction of TCA cycle and energy metabolism pathways. Integrating transcriptomic and metabolomic data revealed significant enrichment of carbohydrate and energy metabolism pathways in the *Yemenite* citron. Additionally, we identified a transcription factor regulatory network that may contribute to juice sac initiation. These findings provide new insights into the molecular processes underlying juice sac initiation and establish a foundation for future research aimed at elucidating its regulatory mechanisms.

**Supplementary Information:**

The online version contains supplementary material available at 10.1007/s00425-026-05008-9.

## Introduction

Citrus is cultivated in over 140 countries, and its production for both the fresh market and the juice industry has continuously increased in recent decades (Spreen et al. 2020). Among angiosperms in the Rutaceae family, citrus belongs to the subfamily Aurantioideae, which is known for its distinctive true berry fruit, termed hesperidium (Esau 1965; Fahn 1990). The hesperidium consists of three layers: the exocarp, which forms the outer, colored skin called the flavedo; the mesocarp, which forms the inner, spongy peel called the albedo; and the endocarp, which comprises two to three layers of inner cells of the albedo adjacent to the locule (Schneider 1968). Following fertilization and fruit set, juice sac primordia begin to develop through anticlinal divisions in endocarp cells and periclinal divisions in the adjoining subepidermal cells (Nii and Coombe, 1988a; Burns et al. 1994; Tisserat et al. 1990). Juice sacs continue to enlarge and fill the locule, which defines the fruit section. Each juice sac is connected to the section wall by a stalk. The epidermal layer covering the section forms a continuous layer surrounding the juice sacs (Tadeo et al. 2020). Most juice vesicles emerge from the dorsal wall of the section, with only a few arising from the side wall. Each section contains two side vascular bundles and one dorsal vascular bundle, and in most cases, juice sacs develop near the vascular bundle (Koch and Avigne 1990). It is generally accepted that the vascular bundles are not directly connected to the juice sacs, and photo-assimilates are mainly transported by diffusion (Sadka et al. 2019). Seeds, when present, develop at the inner junction of the sections.

Given its economic importance, the primary and secondary metabolism of citrus fruit is well characterized. Moreover, numerous studies have described the transcriptomes, proteomes, and metabolomes of juice sacs in various cultivars during different stages of fruit development (Katz et al. 2007, 2010, 2011; Gmitter et al. 2012; Yu et al. 2012; Ibáñez et al. 2014; Ding et al. 2015; Lin et al. 2015; Perotti et al. 2015; Zheng et al. 2016). The integration of these studies has resulted in a relatively good understanding of the metabolic pathways contributing to fruit taste, flavor, and color, as well as to developmental processes and responses to biotic and abiotic cues (Tadeo et al. 2008). However, to the best of our knowledge, the control of juice sac initiation from endocarpal tissue remains poorly studied. Compared to other berry-type fruits, a question arises regarding the unique properties of the citrus endocarp that enable juice sac initiation and development. Citron (*Citrus medica* L.) is one of the earliest cultivated citrus species and, along with pummelo (*C. grandis*) and mandarin (*C. reticulata*), is considered a progenitor of other varieties, such as lemon and lime (Karp and Hu 2018). Among citron cultivars, two— “*Buddha Fingers*” and “*Yemenite*”—lack juice vesicle initiation. However, *Buddha Fingers* lacks locules, and juice vesicles can sometimes be detected between the “fingers.” This suggests that the potential to initiate juice vesicles exists in this cultivar, but the absence of locules prevents their development. In contrast, *Yemenite* citron maintains normal fruit morphology, including exocarp, mesocarp, endocarp, and seed-containing locules (Karp and Hu 2018). Thus, it can be concluded that the absence of juice sacs in this cultivar likely results from a developmental abnormality in the endocarp cells.

By comparing *Yemenite* (Citrus medica var. Etrog) with the juice sac-containing *Calabria* citron (*Citrus medica* var. vulgaris), we aimed to study and understand the regulation of juice sac initiation. Recently, we showed that juice sac primordia could be detected one-week post-anthesis at the dorsal wall of the locule in *Calabria*, becoming clearly visible two weeks post-anthesis (Assili et al. 2024). In contrast, *Yemenite* citron did not produce any juice vesicles. A comparative hormonal analysis of whole ovaries from closed flowers, flowers at anthesis, and fruitlets one and two weeks after anthesis in *Yemenite* and *Calabria* citrons revealed that the most abundant hormones—abscisic acid (ABA), gibberellin A4, indole-3-acetic acid, isopentenyladenine, jasmonic acid, and zeatin riboside—were present at higher levels in *Yemenite* than in *Calabria*. Furthermore, transcriptomic analysis of endocarp cells showed that changes in the expression of ABA metabolic and related genes were consistent with changes in hormone levels in ovaries and fruitlets. This indicates that ABA levels in endocarp cells follow a pattern similar to that in ovaries and fruitlets. While this provides only correlative data, it may indicate that the hormone may negatively regulate the initiation of juice sac primordia in *Yemenite* citron (Assili et al. 2024). Given these results, we assumed that additional comparative analyses of endocarp and other ovary and fruitlet tissues from *Yemenite* and *Calabria* citrons could help identify further regulatory processes associated with juice sac initiation.

In this work, we aimed to further investigate the process of juice sac initiation by performing transcriptomic analysis of endocarp tissue from *Calabria* and *Yemenite* citrons, including the use of Weighted Gene Co-Expression Network Analysis (WGCNA), along with non-targeted metabolomics of ovaries and fruitlets at various developmental stages, both before and during juice vesicle initiation.

## Material and methods

### Plant material

Samples of ovaries and fruitlets were collected from *Calabria* citron (*Citrus medica* var. Vulgaris), which contains juice sacs, and *Yemenite* citron (*Citrus medica* var. Etrog), which lacks juice sacs. The samples were obtained from commercial orchards of adult trees located in the central coastal region of Israel. Three trees were randomly selected based on their inner position within the row. Flowers and fruitlets (n = 25) of similar size were collected from the southeast side of each tree, with each tree considered as one biological replicate.

### Transcriptomic analysis

Samples of the two to three innermost cell layers of the ovary/fruitlet wall, adjacent to the locule, of closed flowers (CF), flowers at anthesis (A), and fruitlets 1 (A1W) and 2 (A2W) weeks following anthesis were collected for RNA extraction with laser capture microdissection (LCM, Zeiss PALM MicroBeam laser microdissection system) as described previously (Martin et al. 2016; Assili et al. 2024). Online Resource 1 presents cross section of Calabria citron and the sampling for LCM. Three replicates of four to five ovaries/fruitlets, containing about 1000–4000 cells of endocarp tissue per sample, were collected into adhesive cap of a collection tube (Adhesive Cap 200 opaque, Zeiss, cat. no. 415190–9181-000). RNA extraction was performed by RNeasy Micro Kit according to manufacturer's instructions (Qiagen, Hilden, Germany). The samples were amplified and subjected to Illumina sequencing at the Nancy and Stephen Grand Israel National Center for Personalized Medicine, The Weizmann Institute of Science, Rehovot, Israel using the INCPM-mRNA-seq. In brief, the poly A fraction (mRNA) was purified from 500 ng of total input RNA followed by fragmentation and generation of double-stranded cDNA. End repair, A base addition, adapter ligation and PCR amplification steps were performed following Agencourt Ampure XP beads cleanup (Beckman Coulter). The libraries were quantified by Qubit (Thermo fisher scientific) and TapeStation (Agilent). Sequencing was executed with a NovaSeq 6000 SP 100 cycles kit, allocating 20M reads per sample (Illumina; single read sequencing). Due to technical difficulty to obtain sufficient cells for the RNA extraction, two replicates were used for the analyses.

Raw reads were subjected to procedures of filtering and cleaning. Illumina adapters were removed from the reads by trimmomatic tool (Bolger et al. 2014), and FASTX Toolkit (http://hannonlab.cshl.edu/fastx_toolkit/index.html, version 0.0.13.2) was used to trim read-end nucleotides with quality scores < 30 (FASTQ Quality Trimmer), and to remove reads with less than 70% base pairs with a quality score ≤ 30 (FASTQ Quality Filter). The clean reads were mapped to the reference genome of orange (Citrus sinensis v2.0_HZAU) using STAR software (Dobin et al. 2013) with an average mapping rate of 93.7%. Gene abundance was estimated using Cufflinks (Trapnell et al. 2010) combined with gene annotations from the plantgarden database (https://plantgarden.jp/en/list/t2711/genome/t2711.G001) and Citrus Pan-Genome to breeding database (https://citrus.hzau.edu.cn). Gene-expression values were computed as fragments per kilobase of exon per million mapped fragments (FPKM). Differential expression analysis was completed using the DESeq2 R package (Love et al. 2014). Genes with an adjusted p-value smaller than 0.05 were considered differentially expressed.

The gene sequences were used as query terms for a search of the NCBI non-redundant (nr) protein database that was carried out with the DIAMOND program (Buchfink et al. 2014). The search results were imported into Blast2GO version 4.0 (Conesa et al. 2005) for Gene Ontology (GO) assignments. Gene ontology enrichment analysis was carried out using Blast2GO program based on Fisher’s Exact Test with multiple testing correction of false discovery rate (FDR). KOBAS 3.0 tool (http://kobas.cbi.pku.edu.cn/kobas3/?t=1) was used to detect the statistical enrichment of differential expression genes in KEGG pathway and Gene Ontology (GO). Cluster analysis of the differentially expressed genes was conducted using Expander 7 software (Ulitsky et al. 2010) with the K-means algorithm (Shamir et al. 2005). The number of clusters was set to 5. In accordance with Feng et al., (2021), BLAST searches with *Arabidopsis* protein sequences were used to identify gene families.

Venn diagrams were generated using Bioinformatics & Evolutionary Genomics (https://bioinformatics.psb.ugent.be/webtools/Venn/), and GO annotation analyses, including REVIGO (Supek et al. 2011), were initiated by AgriGOv2 (http://systemsbiology.cau.edu.cn/agriGOv2/). KEGG analysis was carried out by MetaboAnalyst 5.0 (https://www.metaboanalyst.ca/home.xhtml). Graphic figures for REVIGO and KEGG analyses were constructed with the R package ggplot. Further, Gene co-expression networks were constructed using the R package WGCNA (v 1.63) (Langfelder and Horvath 2008). A total of 2057 differentially expressed genes (DEGs) with an average FPKM from the tested samples were used to perform the co-expression network analysis by weighted correlation network analysis (WGCNA).

Transcriptomic data was deposited in NCBI BioSample and BioProjcet under the following accession numbers (the first number in Biosample and the second number in Bioproject): C_A (SAMN56320921, PRJNA1431751), C_A1W (SAMN56320922, PRJNA1431751), C_A2W (SAMN56320923, PRJNA1431751) C_CF (SAMN56320924, PRJNA1431751), Y_A (SAMN56320925, PRJNA1431751), Y_A1W (SAMN56320926, PRJNA1431751), Y_A2W (SAMN56320927, PRJNA1431751), Y_CF (SAMN56320928, PRJNA1431751).

### Quantitative Real-Time PCR

The expression of selected genes was quantified in pooled plant material containing ovaries and fruitlets of CF, A, A1W, and A2W, as well as in young, fully expanded leaves, using the StepOnePlus system (Applied Biosystems) according to the manufacturer’s instructions. The primers used in this study are listed in Online Resource 2, including the *Citrus sinensis* gene β-actin (*Cs1g05000*), which served as the reference gene.

### Conventional PCR

As one of the identified transcription factors, TF4 (Cs1g08000) could not be detected by quantitative Real-time PCR, conventional PCR was performed using 2 × PCRBIO Taq Mix Red (PCR Biosystems Inc., East Hartford, USA) and specific primers (Online Resource 2) according to manufacturer's instructions. PCR reaction included initial denaturation at 95 °C for 3 min, 50 cycles of denaturation at 95 °C for 30 s, annealing at 58 ^0^C for 30 s and extension at 72 ^0^C for 60 s, followed by final extension at 72 °C for 10 min. The products were separated on 1.7% TAE agarose gel with pUC19 DNA/MspI (HpaII) Marker (Thermo Fisher Scientific, Warrington, UK).

### Metabolomic analysis

The metabolomic analysis was conducted on the ovary wall/fruitlet mesocarp and endocarp, excluding the exocarp, the central tissue connecting the locules/sections and the ovules/seeds. Three replicates of fresh plant material (CF, A, A1W and A2W), were lyophilized, and 24 samples containing about 30 mg dry weight, subjected to untargeted Primary metabolomics analysis, by ALEX-CIS GCTOF MS, at the West Coast Metabolomics Center, UC Davis, CA, USA, as described (https://metabolomics.ucdavis.edu/core-services).

### Statistical analyses

The statistical test used for analysing the metabolites; Principal Component Analysis (PCA), hierarchical clustering (Consolation plot) and Two-way ANOVA test, were performed using MetaboAnalyst-5.0 and JMP 14.

### Joint-Pathway analysis

To integrate transcriptomic and metabolomic datasets, we employed the Joint Pathway Analysis module of MetaboAnalyst to obtain a comprehensive view of metabolic and transcriptional alterations, enabling the identification of biologically relevant pathways co-regulated at both the transcript and metabolite levels. This module allows simultaneous analysis of gene expression and metabolite abundance data within the context of KEGG metabolic pathways. Significantly altered genes (adjusted p-value < 0.05) from the transcriptome data and differentially abundant metabolites were uploaded. The analysis was performed using the hypergeometric test for pathway enrichment and the degree centrality method for topological pathway impact analysis, both of which are default settings in the tool. As KEGG does not currently provide a species-specific database for *Citrus medica* or any other *Citrus* species, we selected *Arabidopsis thaliana* as the reference organism for pathway mapping. This enabled pathway associations to be inferred based on conserved metabolic and regulatory processes across plant species.

## Results

The source tissue for the juice sacs (JSs) is the endocarp, defined as the two to three inner cell layers of the pericarp, which in citrus fruit corresponds to the albedo, the white inner peel (Online Resource 1). Previously, we presented a developmental analysis of *Calabria* ovaries from fruitlet samples at the stages of closed flower (CF), flower at anthesis (A), fruitlets 1 weeks flowing anthesis (A1W), and fruitlets 2 weeks flowing anthesis (A2W), which showed that JS primordia appeared at A1W on the dorsal wall of the locule and became clearly visible at A2W (Assili et al. 2024). In contrast, a similar analysis of *Yemenite* ovaries confirmed the absence of JS initiation in this citron variety.

### Comparative transcriptomic analysis of *Calabria* and *Yemenite* endocarpal cells

The two to three innermost cell layers of the mesocarp, adjacent to the locule and defined as the endocarp, were dissected by Laser Capture Microdissection (LCM) during the four analysed developmental stages—CF, A, A1W, and A2W—of *Calabria* and *Yemenite* citrons (Assili et al. 2024). RNA extracted from these samples was amplified and subjected to deep sequencing. A total of 29,656 genes were successfully mapped (Online Resource 3). Except for *Calabria* samples at stage A, replicates from the same tissue clustered together (Online Resource 4). Samples of *Yemenite* citron at stages CF and A clustered together, unlike the corresponding stages of *Calabria* citron. Stage A1W samples from both cultivars co-clustered, while stage A2W samples were relatively distant from each other.

**Fig. 1:**
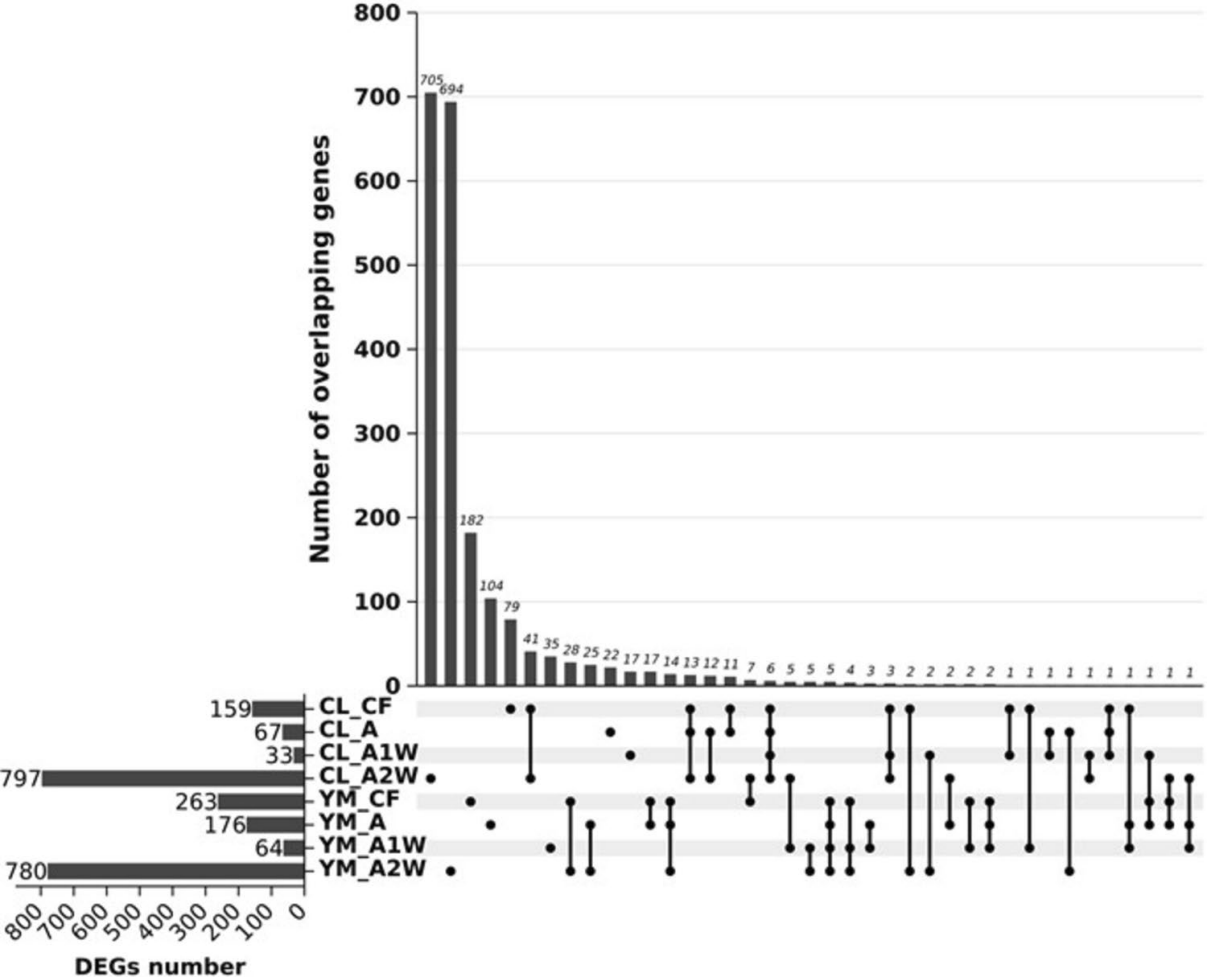
UpSet diagram depicting differentially expressed genes (DEGs). The upper plot illustrates the total count of DEGs in Calabria and Yemenite citrons at CF, A1W, A2W, and A3W stages. Count of overlapping DEGs among each cultivar and stage in shown in the lower panel

When comparing both cultivars at each stage separately, we identified 2,339 differentially expressed genes (DEGs) with at least a twofold change (2FC) and an adjusted p-value ≤ 0.05. Among these DEGs, 219 were significantly different in more than one developmental stage (Online Resource 5 and Fig. 1). In association with the increased number of juice sac primordia during stage A2W (Assili et al. 2024), most of the DEGs—1,577 (797 in *Calabria* and 780 in *Yemenite*)—were found at stage A2W, with most of them being unique: 705 in *Calabria* and 694 in *Yemenite*. Stages CF, A, and A1W displayed 422 (159 in *Calabria* and 263 in *Yemenite*), 243 (67 in *Calabria* and 176 in *Yemenite*), and 97 (33 in *Calabria* and 64 in *Yemenite*) DEGs, respectively, with many unique to each stage (Fig. 1).

**Fig. 2:**
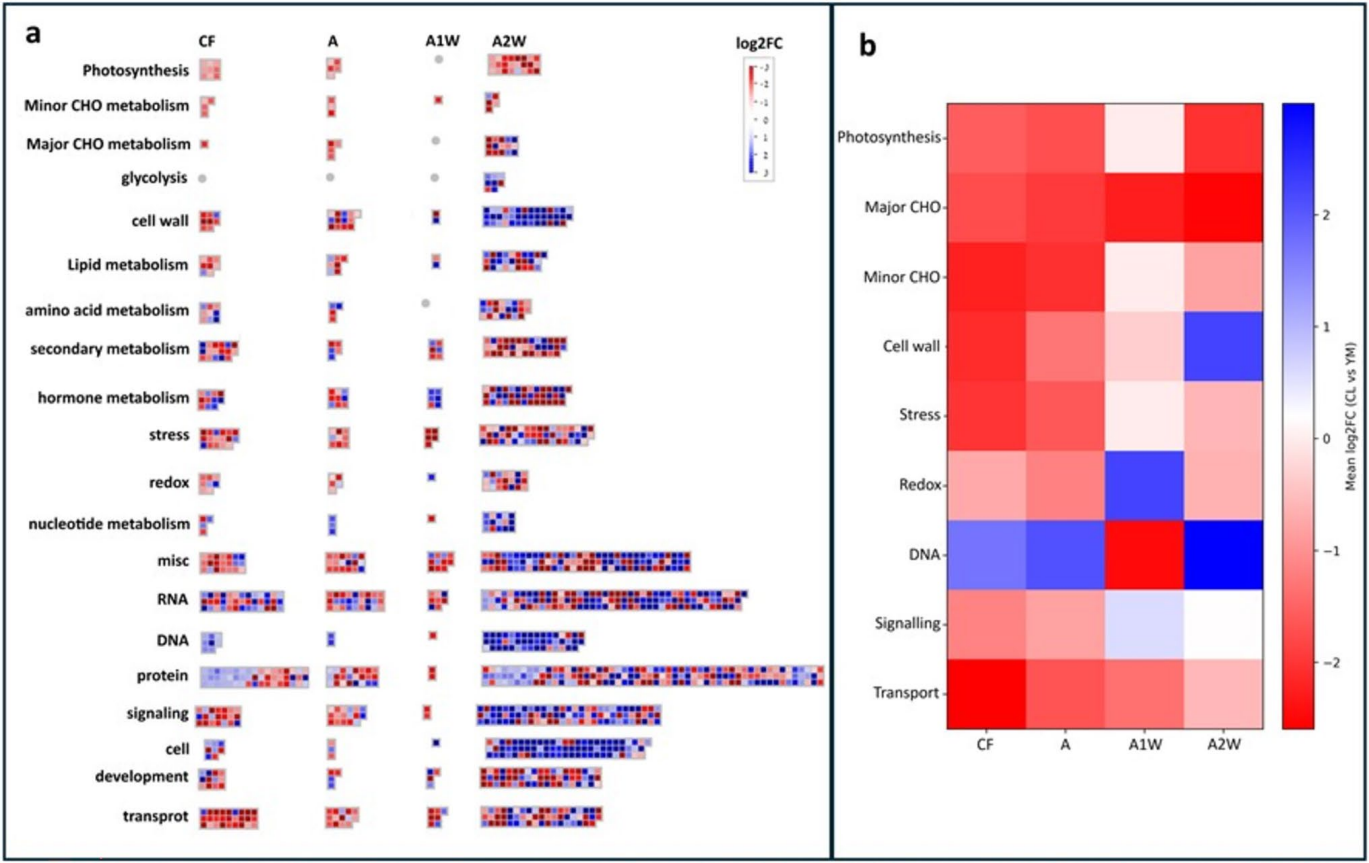
MapMan analysis of transcriptomic data showing differential processes between Calabria and Yemenite at four developmental stages (CF, A, A1W and A2W). The colour blue represents up regulated transcripts (Calabria) and the colour red represents down-regulated transcripts (Yemenite)

MapMan analysis of the DEGs revealed the following patterns: (1) photosynthesis and major and minor carbohydrate metabolism processes were enriched in *Yemenite* citron; (2) cell wall-related processes were enriched in *Yemenite* citron during stages CF and A but showed clear enrichment in *Calabria* citron at stage A2W; (3) stress- and redox-related processes were enriched in *Yemenite* citron; (4) DNA-, signalling-, and cell cycle-related processes were enriched in *Calabria* citron, particularly during stage A2W; (5) transport processes were enriched in *Yemenite* citron, especially during stages CF, A, and A1W (Fig. 2 and Online Resource 6–9).

DEGs mapped to carbohydrate metabolism included 20 genes, which, as noted above, were induced in *Yemenite* citron (Online Resource 9). These included two BETA-AMYLASE-like genes (Cs9g04980, orange1.1t03470) involved in stress-induced starch metabolism.

(Kaplan and Guy 2004; Fan et al. 2024; Berndsen et al. 2024); five raffinose family genes (Cs3g15460, Cs5g05900, Cs4g05490, Cs9g12460, Cs4g07840) associated with stress responses (Yan et al. 2022; Sanyal et al. 2023); and two genes related to trehalose biosynthesis (Cs4g02730, Cs2g17640), which modulate cell growth (Morales-Herrera et al. 2023; Griffiths et al. 2025).

In the process of cell wall formation and modification, 41 genes were detected, with most showing increased expression in *Calabria* compared to *Yemenite* citron (Online Resource 9). These included three EXPANSIN genes (Cs7g03630, Cs8g18640, Cs6g15990) and a COBRA-like gene (Cs5g12210) involved in cell wall loosening (Cosgrove 2000, 2024; Samalova et al. 2022); three genes related to cellulose synthesis (Cs7g07050, Cs7g05150, Cs4g08560); and nine genes encoding pectate lyases and polygalacturonases (Cs8g11330, Cs7g21940, orange1.1t02511, Cs7g08820, Cs9g03730, Cs9g01630, Cs5g03170, Cs7g08600, Cs1g12730) responsible for cell wall degradation (Lu et al. 2025; Anderson and Pelloux 2025).

In DNA- and cell cycle-related processes, 123 genes were detected, with many showing increased expression in *Calabria* compared to *Yemenite* citron (Online Resource 9). These genes were related to DNA replication and repair, chromatin phase separation, cell cycle regulation, and cytokinesis.

In stress response processes, 53 genes were detected, with most showing increased expression in *Yemenite* compared to *Calabria* citron (Online Resource 9). Among them, we identified nine heat-shock-related genes and three drought-stress-related genes.

### Changes in the expression of transcription factors (TFs)

Assuming that one or more regulatory pathways were altered in *Yemenite* compared to *Calabria*, we next analysed differentially expressed transcription factors (TFs). The transcriptome analysis identified 122 differentially expressed TFs, representing on average about 6% of the total DEGs (Online Resource 8). Most of these TFs belonged to the following gene families: leucine zipper motif (ZIP), Ethylene Responsive Factor (ERF), Homeodomain leucine zipper (HD-ZIP), MADS-box (MADS), NAC-domain transcription factors (NAC), WUSCHEL-related homeobox (WOX), WRKY-domain transcription factors (WRKY), and MYB-domain transcription factors (MYB).

**Fig. 3:**
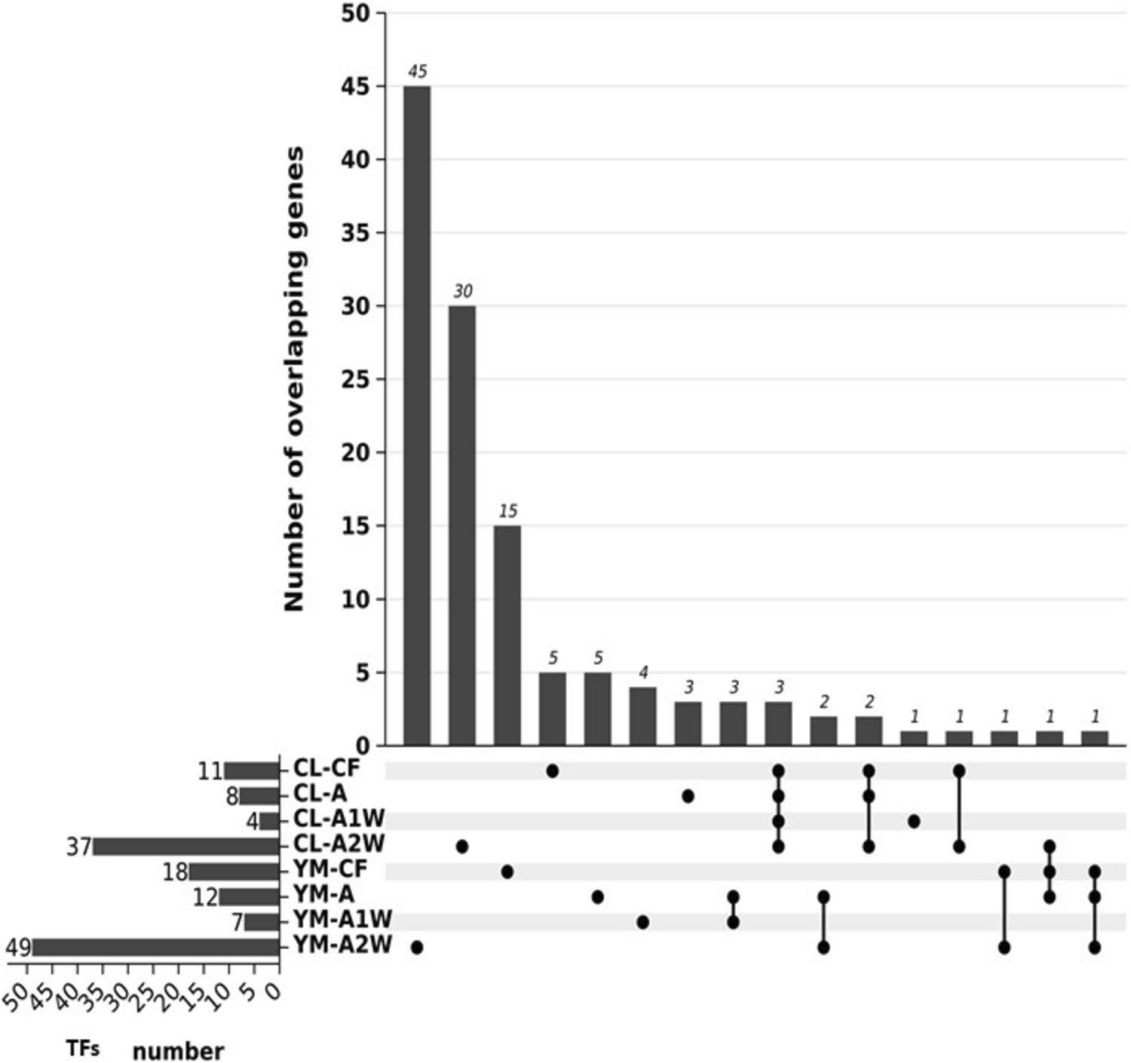
UpSet diagram depicting differentially expressed transcription factors (TFs). The upper plot illustrates the total count of TFs in Calabria and Yemenite citrons at CF, A1W, A2W, and A3W stages. Count of overlapping TFs among each cultivar and stage in shown in the lower panel

**Fig. 4:**
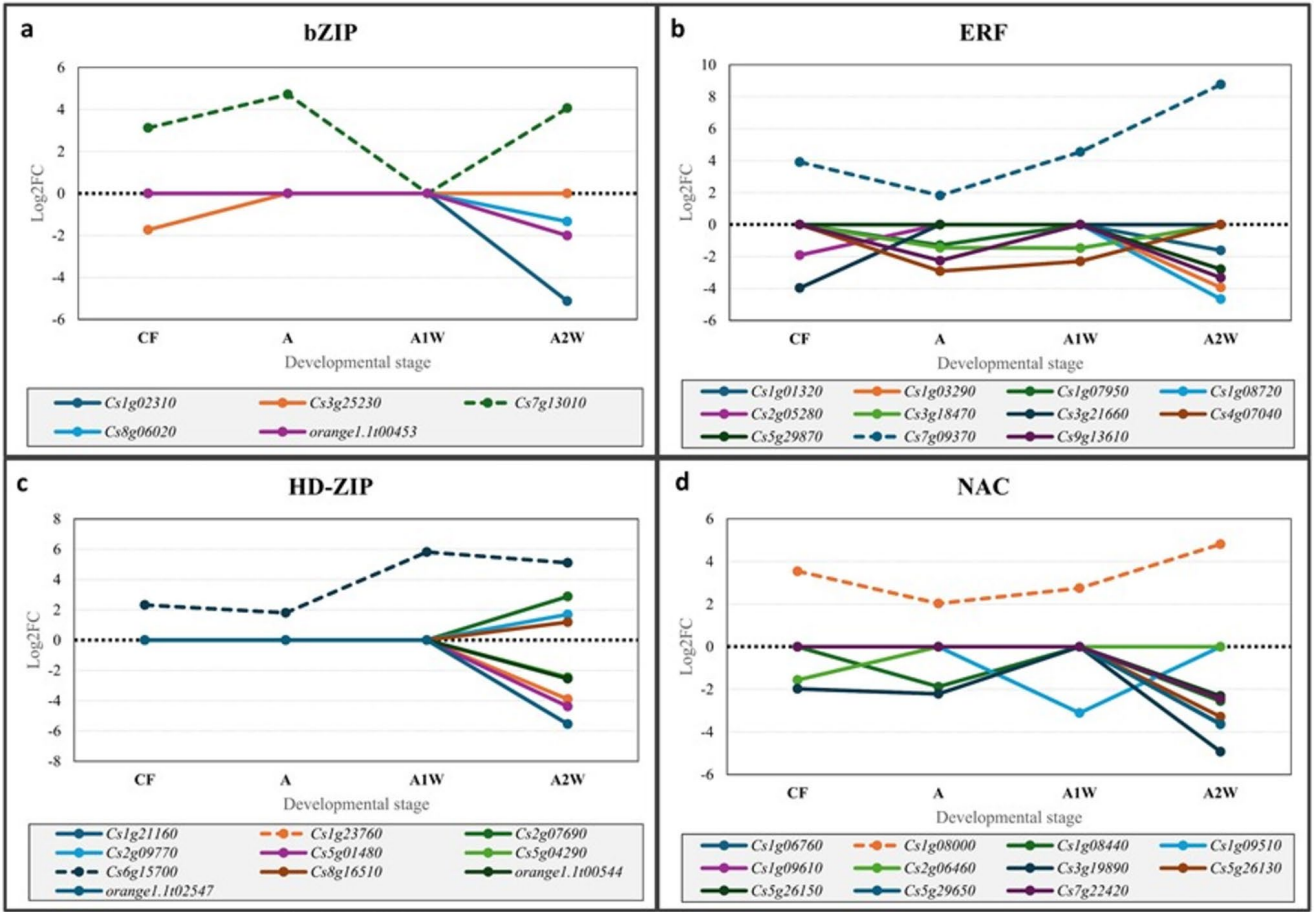
Log 2 of fold change ratios (Calabria/ Yemenite) of differentially expressed transcription factors genes presented by genes families at four developmental stages, CF, A, A1 & A2, as indicated. The following families are shown: basic leucine zipper (bZIP) proteins, Ethylene Responsive Factor (ERF) proteins, Homeodomain-leucine zipper (HD-ZIP), MADS-box transcription factors (MADS), NAC transcription factors (NAC), WRKY transcription factors (WRKY), WUSCHEL − related homeobox (WOX) and MYB transcription factors (MYB)

Most of the detected TFs—108 in total—showed cultivar- and stage-specific expression, with the majority expressed during stage A2W (Fig. 3). Only five TFs were expressed in three or four developmental stages, belonging to the ZIP, ERF, HD-ZIP, and NAC families. Notably, four of these TFs showed expression trends opposite to those of other members of their respective families; while most family members displayed higher expression in *Yemenite*, these four TFs were induced in *Calabria* compared to *Yemenite* (Fig. 4). Annotation of these TFs to Arabidopsis indicated potential roles in regulating tissue formation (detailed below). These included the bZIP gene PERIANTHIA (PAN) (Cs7g13010, TF1), the ERF gene BOLITA/DRN/ESR2 (Cs7g09370, TF2), the HD-ZIP gene LATE MERISTEM IDENTITY1 (LMI1) (Cs6g15700, TF3), and the NAC gene CUP-SHAPED COTYLEDON 2 (CUC2) (Cs1g08000, TF4).

**Fig. 5:**
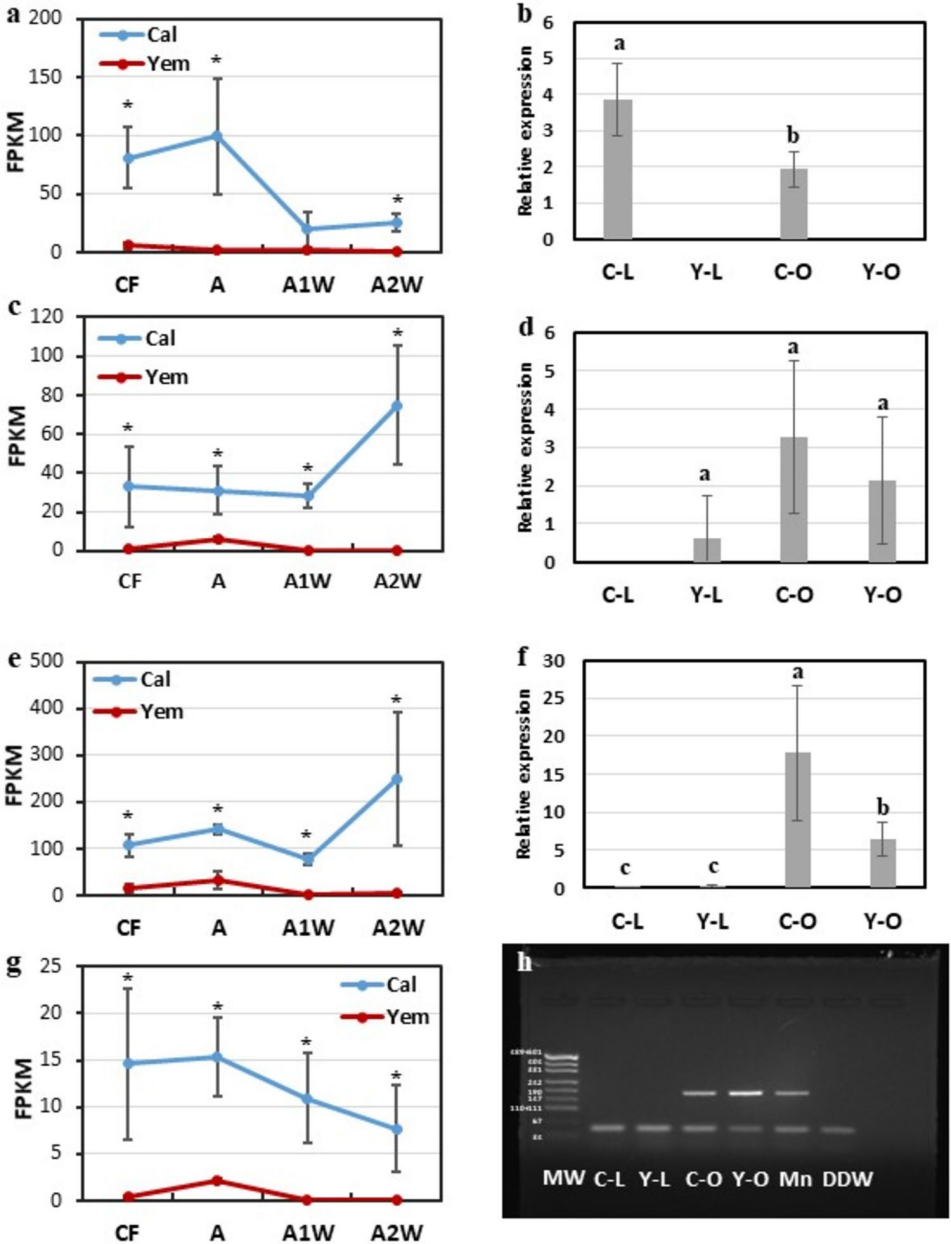
Expression analyses of TF1, TF2, TF3 and TF4 in various tissues. FPKM values of (**a**) TF1 (Cs7g13010, PAN-LIKE), **c** TF2 (Cs7g09370, BOLITA-like/DRNL-like), **e** TF3 (Cs6g15700, LMI1-like) and (**g**) TF4 (Cs1g08000, CUC2-like) in endocarp of Calabria and Yemenite citrons, at four developmental stages (CF, A, A1W and A2W), as indicated. The relative expression of (**b**) TF1, **d** TF2 and (**f**) TF3 on Calabria citron leafs (C-L), Yemenite citron leafs (Y-L), Calabria citron ovaries (C-O), Yemenite citron ovaries (Y–O) and mandarin orange (Citrus reticulata) leafs (Mn). Lowercase letters and * represent statistically significant differences between the corresponding developmental stages (P < 0.05) analysed using one-way ANOVA. Agarose gel electrophoresis (2%) of PCR product of TF4 primers on C-L, Y-L, C-O, Y–O, mn and Deuterium-depleted water (DDW) (**h**)

The FPKM values of these four TFs were very low in endocarp cells at all analyzed developmental stages in *Yemenite* citron compared to *Calabria* (Fig. 5). TF1 showed relatively high FPKM values in endocarp cells during stages CF and A in *Calabria* citron, which were reduced in later stages (Fig. 5a). Its transcripts were detectable in *Calabria* leaves and whole ovaries but were below detection in *Yemenite* organs (Fig. 5b).

In *Calabria* citron, TF2 and TF3 showed relatively constant expression in endocarp cells during CF, A, and A1W stages, with a 2.5- to threefold increase at A2W (Fig. 5c, e). These two TFs were undetectable in *Calabria* leaves, while TF2 showed low expression in *Yemenite* leaves. Both TFs were expressed in whole ovaries/fruitlets of both cultivars (Fig. 5d, f); however, TF2 displayed similar expression levels in both cultivars, whereas TF3 transcript levels were about threefold lower in *Yemenite* citron compared to *Calabria*.

TF4 expression in endocarp cells was constant during CF, A, and A1W stages in *Calabria* citron and was reduced by about twofold at A2W (Fig. 5g). This gene was not detectable by qPCR in leaves or whole ovaries of either cultivar. However, conventional PCR with a high number of cycles detected its transcripts in ovaries/fruitlets of both cultivars (Fig. 5h).

In addition to these four TFs, we identified another MYC-like bHLH transcription factor, MYC67 (orange1.1t0057), which was induced in *Calabria* citron at three of the four analysed developmental stages (Online Resource 10).

### Weighted Gene Co-Expression Network Analysis (WGCNA) for differentially expressed genes (DEGs)

**Fig. 6:**
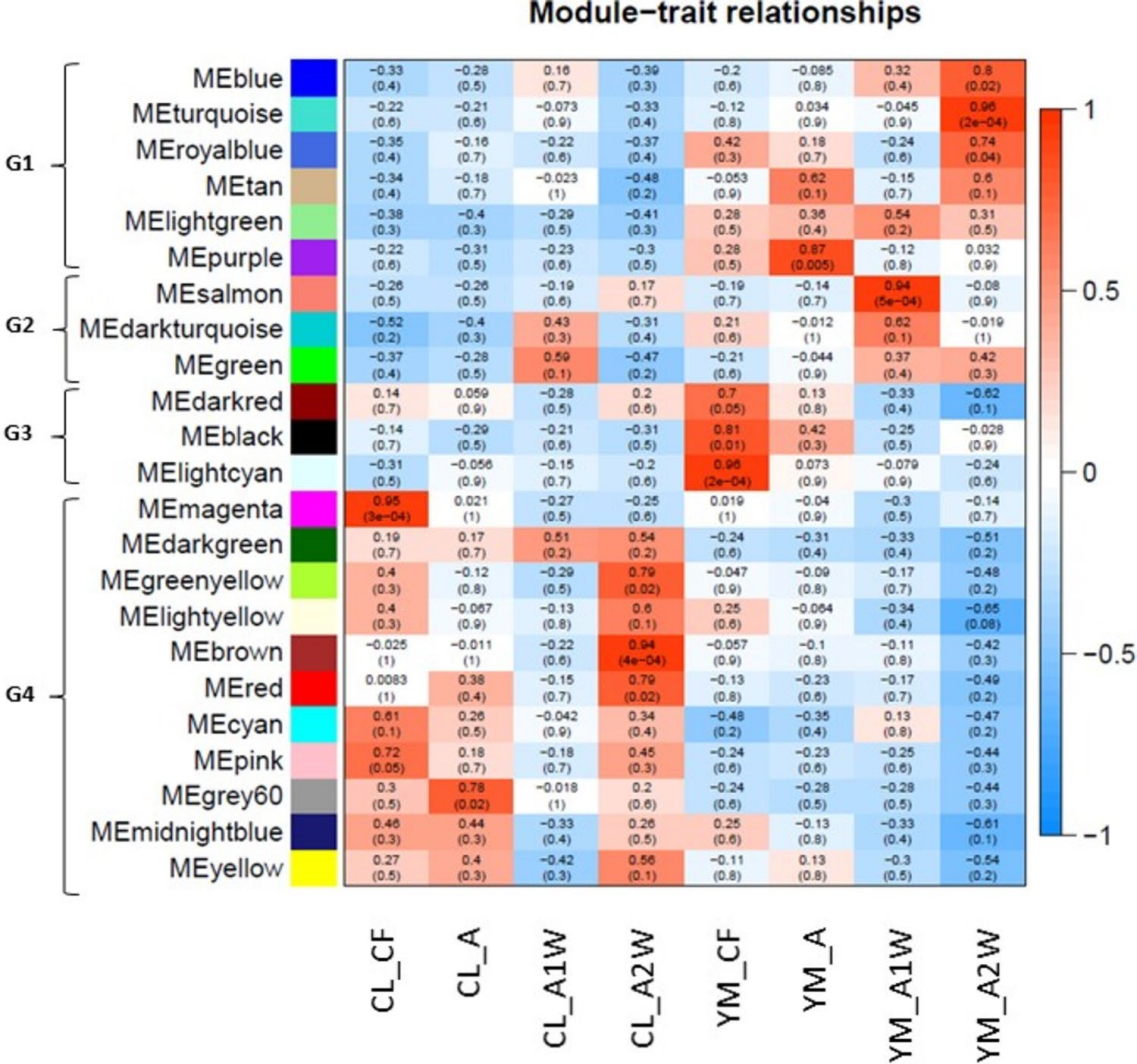
Heatmap showing the module-trait relationships by weighted gene co-expression network analysis (WGCNA) of genes between two cultivars of citron, Calabria and Yemenite, at four developmental stages, CF, A, A1W and A2W. Each row corresponds to a module and each column corresponds to different combinations of cultivar X stage. The top and bottom number in each cell indicate the correlation coefficient between the module and combination, p-value of the test, respectively. Modules classified into four groups: G1, G2, G3 and G4

WGCNA was used to cluster highly correlated genes into networks (modules) and to identify potential key regulatory genes that may act as drivers of relevant processes. A total of 2,057 DEGs were clustered into 23 modules with varying hierarchical relationships (Online Resources 11 and 12). These modules were classified into four groups (Fig. 6). Group 1 (G1) included six modules—Blue, Turquoise, Royal Blue, Tan, Light Green, and Purple—containing DEGs induced in *Yemenite* citron, particularly during stage A2W. Group 2 (G2) included three modules—Salmon, Dark Turquoise, and Green—containing DEGs induced in both *Calabria* and *Yemenite* citrons, mainly during stage A1W. Group 3 (G3) included three modules—Dark Red, Black, and Light Cyan—containing DEGs induced in *Yemenite* citron during stage CF. The largest group, Group 4 (G4), included ten modules—Dark Green, Green Yellow, Light Yellow, Brown, Red, Cyan, Pink, Grey60, Midnight Blue, and Yellow—containing DEGs induced in *Calabria* citron at various stages.

Among the modules, Blue and Turquoise of G1 contained the largest sets of genes, with 235 and 339 DEGs, respectively. In G4, the Brown and Yellow modules contained 176 and 151 DEGs, respectively (Online Resource 11). Other modules contained fewer than 100 DEGs.

**Fig. 7:**
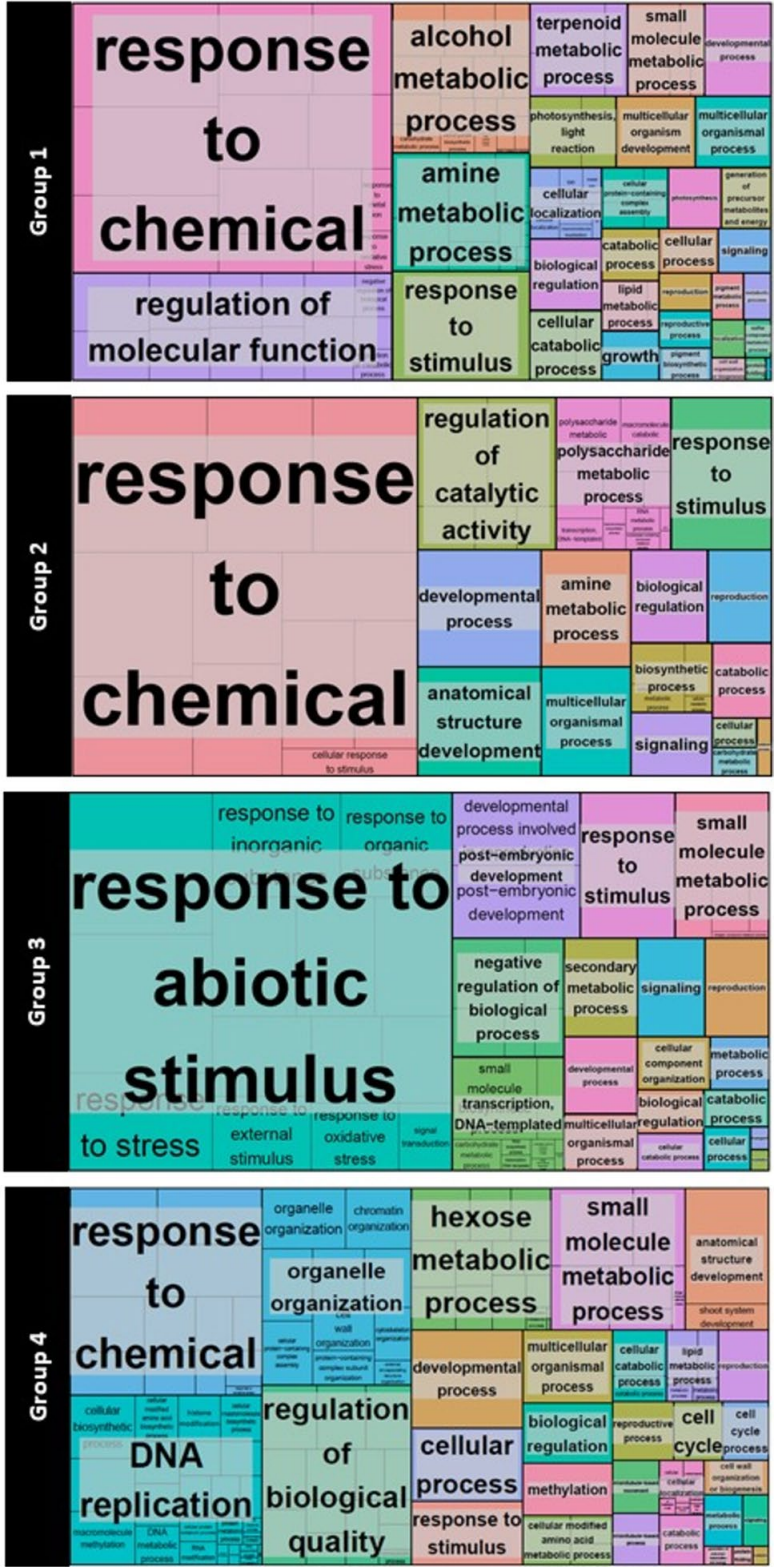
The representatives Visualization of GO enrichment analysis of the genes on Groups 1–4, classified following WGCNA analysis (Fig. 5) by REVIGO. Each rectangle is a single cluster representative. Clusters are joined into ‘superclusters’ of loosely related terms, visualized with different colours. Size of the rectangles was adjusted to reflect the p-value of the GO term

Gene Ontology (GO) analysis of each group showed distinct enrichment patterns (Fig. 7). G1, comprising modules induced in *Yemenite* citron, was enriched in processes related to chemical responses, regulation of molecular function, and metabolism of alcohol, amine, and terpenoids. G2, containing modules induced in both cultivars during stage A1W, was enriched in processes related to chemical responses, developmental processes, and anatomical structure development. G3, containing modules induced in *Yemenite* citron during stage CF, was enriched in responses to abiotic stimuli and developmental processes, particularly post-embryonic development. G4, which included modules induced in *Calabria* citron, was enriched in DNA replication, chromatin organization, cell cycle, organelle organization, multicellular organization, and small molecule and hexose metabolic processes.

Juice sac primordia are produced through anticlinal and periclinal divisions of endocarp cells. The GO analysis of G4, which included modules induced in *Calabria*, revealed enrichment of processes related to DNA replication and the cell cycle. To identify highly interconnected hub genes that may function as key regulators within each module, we constructed gene co-expression networks using WGCNA and Cytoscape (Shannon et al., 2003) (Online Resource 13).

Notably, the Light Cyan module of G3 and the Grey60 and Red modules of G4 had the highest proportions of TFs, with 12%, 14%, and 12% of DEGs in each module, respectively, compared to an average TF abundance of 6% among all DEGs (Online Resource 11). As noted above, G4 included modules induced in *Calabria* citron across all analysed stages. It contained 890 genes, including 51 TFs distributed among 10 modules (Online Resource 11). Among these 51 TFs, 20 had direct edges with at least 50% of the other genes within their respective modules (Table 1). Three of the TFs identified earlier—TF1, TF2, and TF3—were included in this group of 21 TFs. TF1 was classified in the Grey60 module, with edges connecting to 55% of the DEGs in that module. TF2 and TF3 were classified in the Red module, with edges connecting to 66% and 57% of DEGs in their module, respectively. TF4 belonged to the Grey module (Table S10), with edges connecting to 49% of the genes in that module.

**Table 1:**
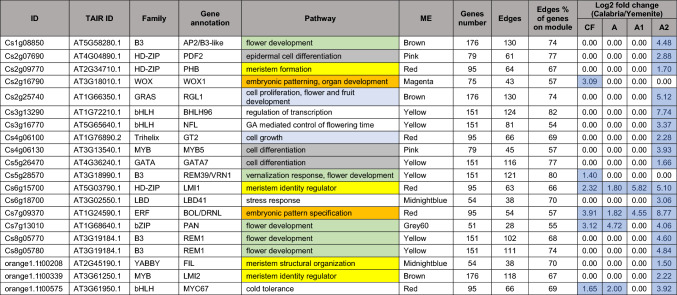
Transcription factors (TFs) with the highest number of edges detected in the WGCNA module network

The table lists gene identifiers (ID), the corresponding Arabidopsis orthologs (TAIR ID), TF family classification, and gene annotation with the associated biological pathway, with identical color code for similar pathways. The column ME indicates the WGCNA module (module eigengene) to which the TF belongs. “Genes number” reports the total number of genes within the module. “Edges” indicates the number of network connections detected for the TF within the module network, and “Edges % of genes on module” reports the proportion of connections relative to the total number of genes in the module. The columns CF, A, A1, and A2 report the log₂ fold change (Calabria/Yemenite) for each condition.

The 20 TFs were induced in *Calabria* compared to *Yemenite* in at least one developmental stage ((Table 1)). Most of these TFs are associated with processes potentially linked to tissue formation and growth, as well as cell differentiation. For instance, Cs2g25740, an ortholog of the *Arabidopsis* gene AT1G66350 encoding the DELLA subfamily GRAS protein RGA-LIKE1 (RGL1), is involved in cell differentiation (Wen and Chang 2002). Several TFs are linked to meristem regulation, including Cs2g09770, an ortholog of AT2G34710 (a HD-ZIP gene encoding PHABULOSA, PHB) (Williams et al. 2005; Müller et al. 2015); orange1.1t00208, an ortholog of AT2G45190, (a YABBY family gene encoding FILAMENTOUS FLOWER, FIL involved in abaxial cell type specification in leaves and fruits) (Lugassi et al. 2010; Bonaccorso et al. 2012); and orange1.1t00339, an ortholog of AT3G61250 (a MYB family gene encoding LATE MERISTEM IDENTITY2, LMI2) (Pastore et al. 2011). Other TFs were associated with flower development, such as Cs1g08850, an ortholog of AT5G58280 (an AP2/B3-like TF encoding a DNA-binding protein) (Zhou et al. 2021; Jiang et al. 2022), and Cs8g05770 and Cs8g05780, orthologs of AT3G19184 (AP2/B3-like TFs encoding REPRODUCTIVE MERISTEM1, REM1) (Luna-García et al. 2024). Additionally, Cs2g16790, an ortholog of AT3G18010 (a WUSCHEL-related homeobox gene encoding WOX1), was linked to embryonic patterning (Haecker et al. 2004).

### Untargeted metabolomics comparison between *Calabria* and *Yemenite* citrons

**Fig. 8:**
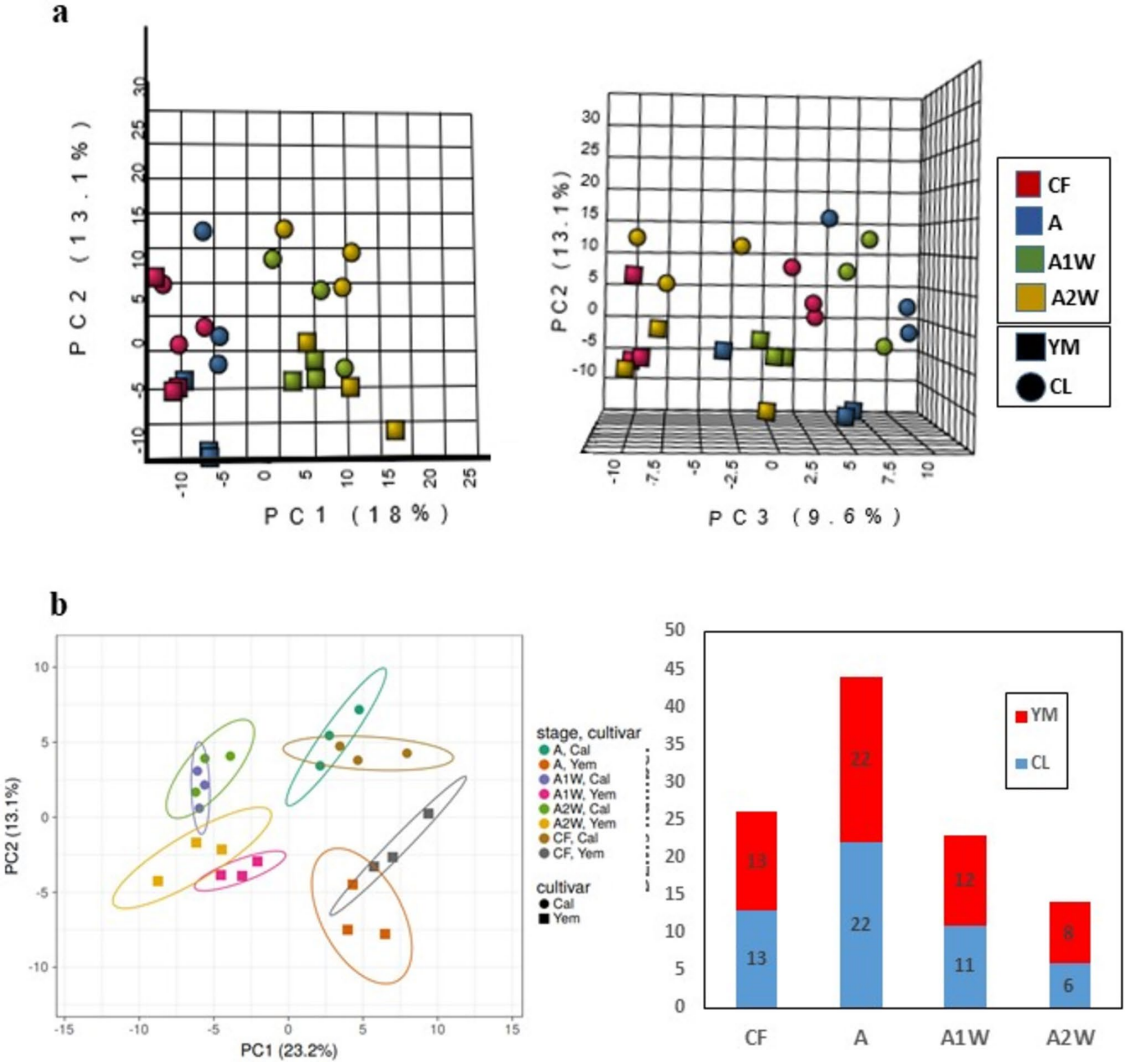
Analysis of the untargeted metabolites by developmental stages and two citron cultivars, Yemenite (YM) and Calabria (CL). Principal component analysis (PCA) of 890 untargeted metabolites: 3D Scores plot between PC1 and PC2, and 3D score plot between PC2 and PC3. **b** PCA of 138 identified metabolites. The explained variances are shown in brackets. **c** Numbers of significantly different metabolites between the Calabria and Yemenite, at the indicated developmental stages (CF, A, A1W and A2W) by One-way ANOVA

In contrast to the transcriptomic analysis, which was conducted in endocarp cells, the metabolomic analysis was performed on whole ovaries and fruitlets, excluding the outer peel (flavedo), the central connecting tissue (diaphragm), and the ovules/seeds (Online Resource 1). Among the 860 detected metabolites, 138 were identified (Online Resource 14). Principal component analysis (PCA) of all detected metabolites showed that, regardless of the four analysed developmental stages, the cultivars were overall separated, especially in PC2 and PC3, but to a lesser extent in PC1 and PC2 (Fig. 8a). Similarly, PCA of the 138 identified metabolites showed a clear separation between cultivars (Fig. 8b). Additionally, stages CF and A were separated from stages A1W and A2W in both *Calabria* and *Yemenite* citrons (Fig. 8a, b). Statistical analysis of the identified metabolites showed that 26, 44, 23, and 14 metabolites displayed significant differences between the two cultivars at stages CF, A, A1W, and A2W, respectively, with an overall even distribution across stages (Fig. 8c).

**Fig. 9:**
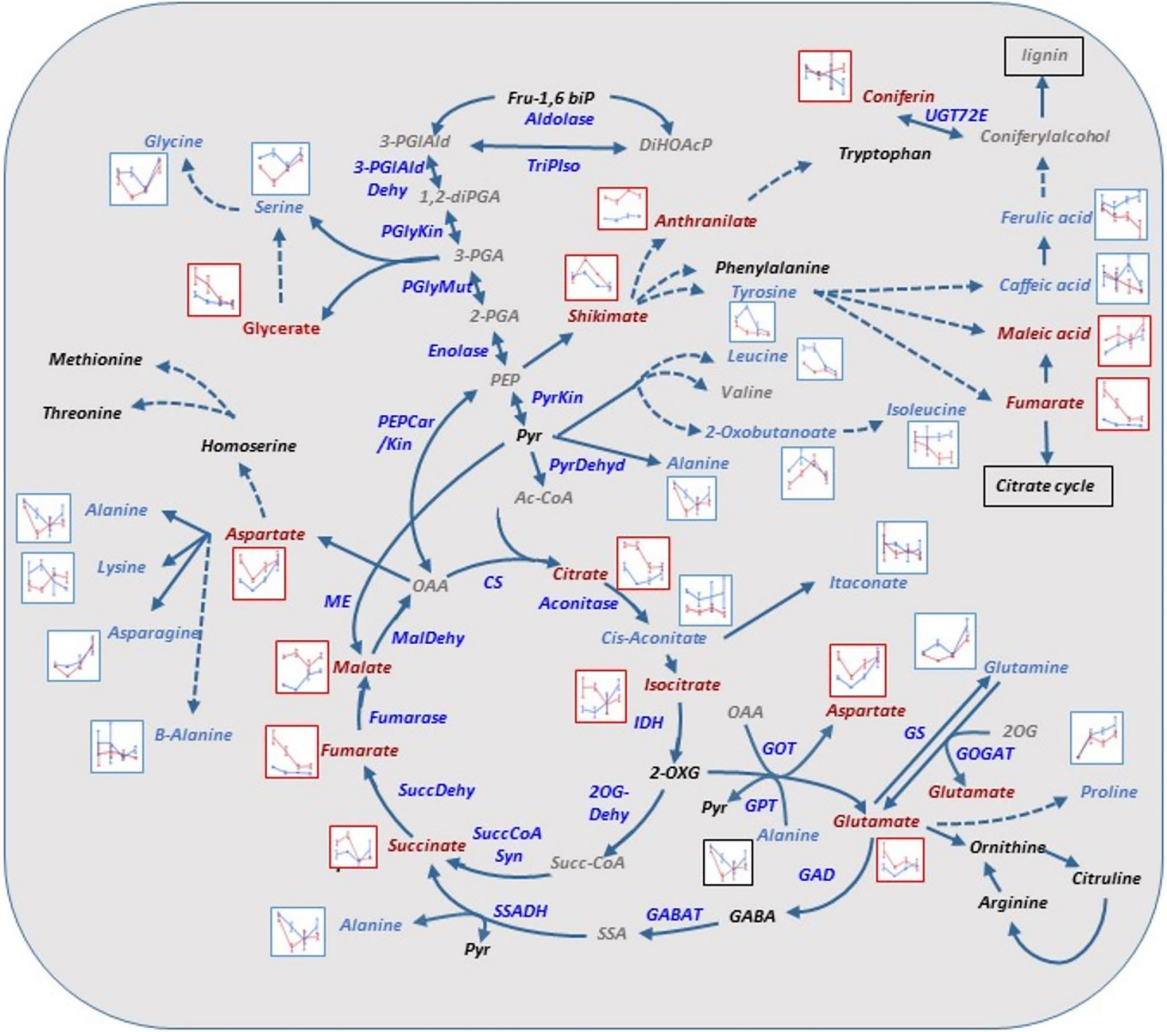
A metabolism scheme of amino acids and organic acids with metabolites showing overall higher levels in Yemenite (red), Calabria (blue), or no change (grey). The average of each metabolite on Calabria and Yemenite at CF, A, A1W, and A2W is shown side to it. Bars indicate standard error

Changes in the levels of primary metabolites (excluding sugars) in *Calabria* and *Yemenite* citrons are schematically summarized in Fig. 9. Most metabolites of the TCA cycle showed higher levels in *Yemenite* compared to *Calabria*, except for *cis*-aconitate, an intermediate of the cycle. However, their developmental patterns differed. Citrate, succinate, and fumarate levels were generally reduced in both cultivars, whereas isocitrate decreased in *Yemenite* but increased in *Calabria*. *Cis*-aconitate levels remained mostly stable in both cultivars.

Most detected amino acids showed higher levels in *Calabria* than in *Yemenite* citron at least at one developmental stage. Aspartate was an exception, with higher levels in *Yemenite*. However, amino acids synthesized from aspartate—alanine, lysine, and asparagine—showed higher levels in *Calabria*, with asparagine induced in both cultivars, alanine reduced overall, and lysine displaying contrasting trends: reduction in *Calabria* and induction in *Yemenite*. Amino acids derived from pyruvate—leucine, isoleucine, and 2-oxobutanoate—and those derived from 3-phosphoglyceraldehyde (3-PGA)—glycine and serine—were generally higher in *Calabria*. Glycine and serine showed identical patterns in both cultivars, with a reduction from CF to A1W followed by re-induction at A2W. While leucine levels decreased in both cultivars, isoleucine remained stable in *Calabria* but decreased in *Yemenite*.

Mixed trends were observed among amino acids derived from 2-oxoglutarate (2-OXG). Alanine, proline, and glutamine showed higher levels in *Calabria*, while glutamate and aspartate were higher in *Yemenite*. Despite these differences, these amino acids followed similar developmental patterns in both cultivars.

Similarly, shikimate and anthranilate showed comparable developmental patterns in both cultivars, although their levels were higher in *Yemenite*. However, tyrosine, derived from shikimate, was more abundant in *Calabria*, despite following a similar pattern in both cultivars, characterized by a gradual reduction across the four developmental stages. Ferulic acid, a precursor in lignin biosynthesis, showed higher levels in *Calabria* during stages A1W and A2W. In *Yemenite*, ferulic acid levels gradually decreased across all stages.

Unlike most amino acids and TCA cycle metabolites, sugars did not show a clear trend (Online Resource 15). Sucrose exhibited opposite patterns: induced in *Yemenite* and reduced in *Calabria*. Glucose 1-phosphate generally showed higher levels in *Calabria*, except at stage A1W, where both cultivars displayed similar levels. Two compounds involved in pectin biosynthesis—galactarate and glucarate—showed higher levels in *Yemenite*. Galactarate levels declined in both cultivars during development, while glucarate remained stable in *Calabria* but increased in *Yemenite* from CF to A, followed by a reduction thereafter.

### Associations between metabolites and their respective metabolic genes

**Fig. 10:**
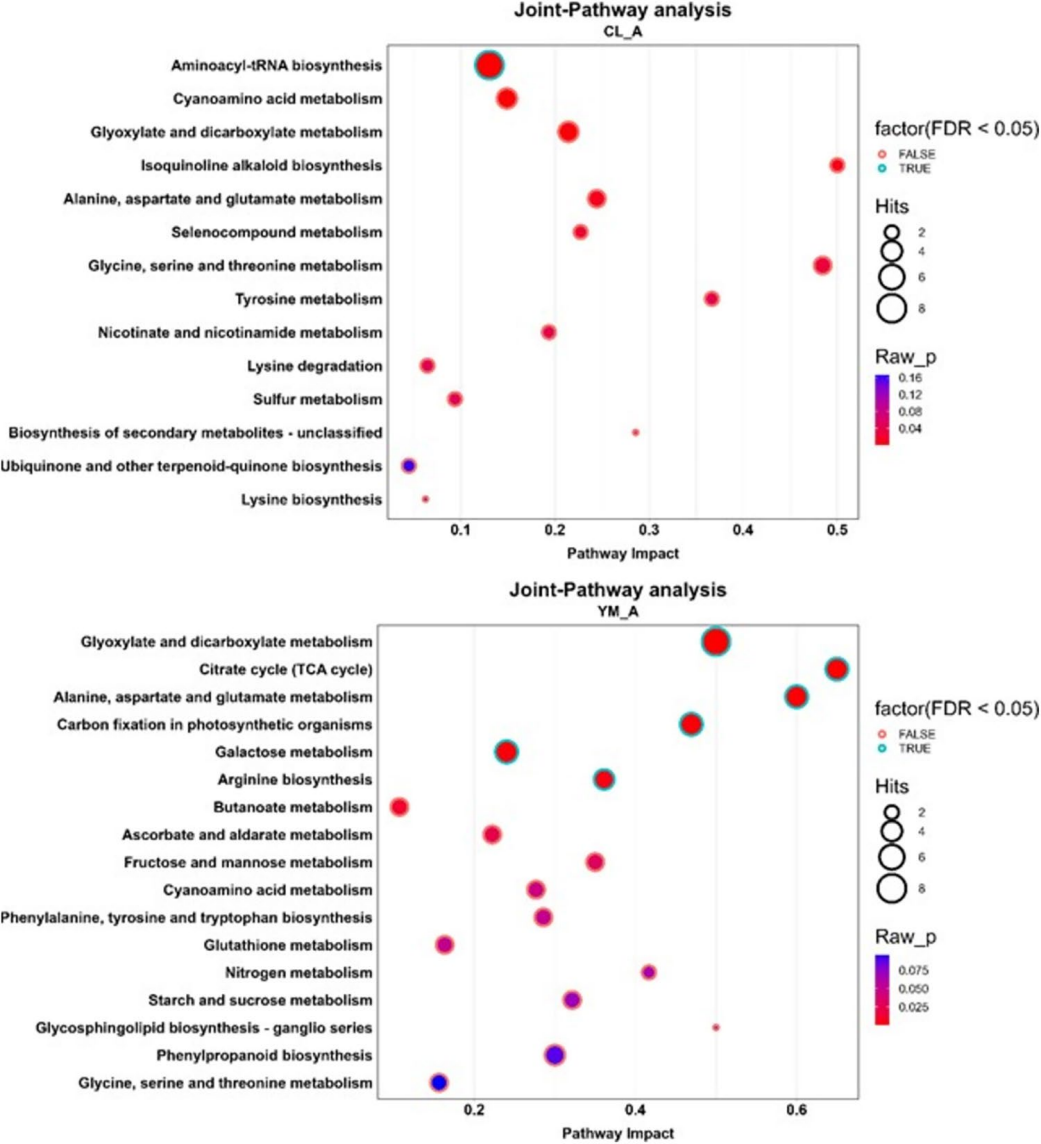
Advanced bubble chart shows significantly enriched pathways based on joint-pathway analysis of differentially expressed genes (DEGs) and metabolite levels, at “A”, by MetaboAnalyst5.0. **a** enriched pathways in Calabria. **b** enriched pathways on Yemenite. The x-axis represents pathway Impact. The y-axis represents the enriched pathways. Fill colour represents enrichment significance by p-value, the border colour represents enrichment significance by FDR, and the size of the bubble represents the number of Hits (DEGs and metabolites) enriched in the pathway

The transcriptomic analysis was conducted in endocarp tissue, whereas the metabolomic analysis was performed on whole ovary and fruitlet tissues. To assess whether the metabolic profiles of whole ovaries and fruitlets reflected metabolic activity in the endocarp, we performed an integrated pathway analysis using the joint pathway analysis module. Figure 10 shows the enrichment results at the anthesis stage for both cultivars, while Online Resources 16 and 17 present the results for stages CF and A1W + A2W, respectively (stages A1W and A2W were combined due to the low number of differential metabolites). Table 2 summarizes the significant pathways enriched in each cultivar or shared between them across the analyzed developmental stages.

**Table 2:**
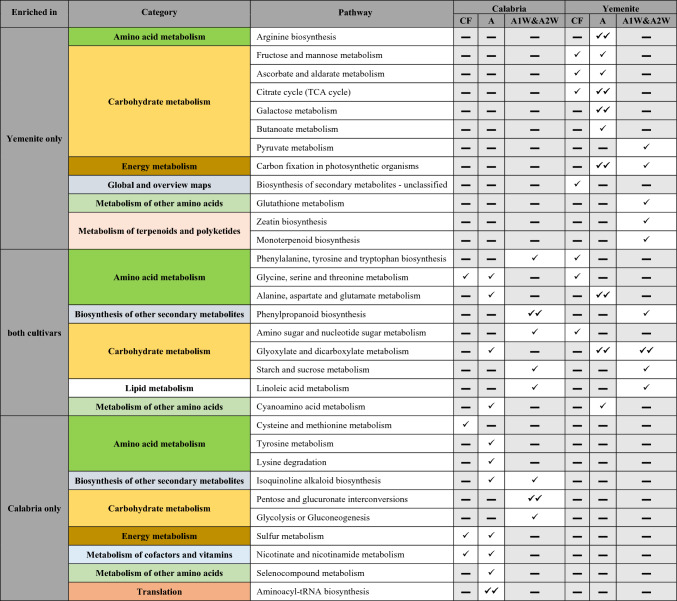
Joint pathway analysis of enriched metabolic processes identified in the two cultivars (Calabria and Yemenite) across the different developmental stages, closed flowers (CF), anthesis (A), and one- and two weeks following anthesis (A1W&A2W)

Pathways are grouped according to their functional KEGG categories and are classified as enriched in Yemenite only, Calabria only, or in both cultivars. For each pathway, enrichment is reported for the corresponding developmental stage in each cultivar. A single symbol (✓) indicates a significantly enriched pathway with p < 0.05, whereas a double symbol (✓✓) indicates enrichment after multiple-testing correction with FDR < 0.05. The symbol (▬) indicates that the pathway is not significantly enriched.

Most of the pathways enriched in *Yemenite* citron at stages CF and A were related to carbohydrate and energy metabolism, with the citrate cycle, galactose metabolism, and carbon fixation pathways showing high significance based on the FDR test during stage A. In *Calabria* citron, only the glyoxylate and dicarboxylate metabolism pathway was enriched among carbohydrate metabolic pathways, while sulphur metabolism was enriched among energy-related pathways.

Among amino acid metabolic pathways, alanine, aspartate, and glutamate metabolism showed significant enrichment in *Yemenite* citron at stage A and was also enriched in *Calabria*. However, many additional amino acid metabolic pathways were enriched in *Calabria* citron at stages CF and A: cysteine and methionine metabolism, tyrosine metabolism, lysine degradation, glycine, serine and threonine metabolism, alanine, aspartate and glutamate metabolism, cyanoamino acid metabolism, seleno-compound metabolism, and aminoacyl-tRNA biosynthesis, with the latter showing FDR < 0.05 at stage A.

At stages A1W + A2W, six pathways related to carbohydrate and energy metabolism were enriched. Three pathways were enriched only in *Calabria* citron: pentose and glucuronate interconversions, glycolysis or gluconeogenesis, and amino sugar and nucleotide sugar metabolism, with pentose and glucuronate interconversions showing high significance based on the FDR test. Two pathways were enriched only in *Yemenite* citron—pyruvate metabolism and glyoxylate and dicarboxylate metabolism—both showing high significance by FDR test. Starch and sucrose metabolisms were enriched in both cultivars. Additionally, the phenylpropanoid biosynthesis pathway was enriched in both cultivars at stages A1W + A2W, with higher significance in *Calabria* citron according to the FDR test.

## Discussion

### Juice sacs initiation in *Calabria* citron

The endocarp consists of the inner portion of the pericarp and part of the locular membrane, along with an epidermal layer accompanied by several layers of adjacent parenchyma cells (Ford 1942). Juice sac (JS) primordia form through anticlinal and periclinal divisions within the epidermal cells, which retain meristematic characteristics (Nii and Coombe 1988b; Burns et al. 1992). Subepidermal cells also divide; however, their daughter cells enlarge and lose meristematic properties. Oblique divisions of epidermal cells, followed by cell enlargement, result in cone-shaped cells that form within the emerging dome structure (Carrillo-López and Yahia 2019; Nii and Coombe 1988b; Ford 1942). Shortly after anthesis, regardless of fertilization, the surface cell layer of the endocarp displays primordial domes of JSs (Assili et al. 2024). The *Yemenite* citron cultivar does not form JSs, possibly due to the absence of anticlinal divisions in the endocarp epidermis or the inability of subepidermal cells to differentiate into enlarged JS cells. The transcriptomic analysis, showing that most DEGs appeared at stage A2W, aligns with primordia emergence; however, the regulatory processes may be initiated at earlier developmental stages.

## Cell proliferation-related processes in *Calabria* citron *versus* stress-related processes in *Yemenite* citron

Results from the transcriptomic analysis, followed by WGCNA, gene annotation (GO), and MapMan, showed that genes in Group 4, enriched during stages CF, A, and A2W in *Calabria* citron (Fig. 6), were associated with responses to chemicals, DNA-related processes (including replication, repair, and chromatin phase separation), cell division, cell cycle regulation, cytokinesis, and organelle organization (Fig. 7). Additionally, processes related to cell wall formation and modification were enriched in *Calabria* at stage A2W, including genes involved in cell wall loosening, such as the EXPANSIN family (Cosgrove 2000), and cell wall degradation, such as pectate lyases and polygalacturonases (Uluisik and Seymour 2020). These findings are consistent with the requirement for cell expansion and proliferation during juice sac initiation. On the other hand, the lack of enrichment of these processes in *Yemenite* citron may suggest suppression of pathways related to cell proliferation and expansion, potentially contributing to its distinct developmental program.

Genes in Group 3, enriched in *Yemenite* citron, particularly at stage CF, were associated with responses to abiotic stimuli, stress, and specifically oxidative stress. In our previous study, we showed that ABA metabolism, signalling, and transport in endocarpal cells, as well as hormonal levels in entire ovaries and fruitlets, were elevated in *Yemenite* compared to *Calabria* citron. ABA is known to suppress vegetative growth by inhibiting cell division and differentiation (De Smet et al. 2003; Shimizu-Sato and Mori 2001; Zhang et al. 2010). Therefore, it is tempting to speculate that ABA may also suppress juice sac initiation. Further, ABA is also closely linked to stress responses and is induced during various stress conditions, promoting the accumulation of reactive oxygen species (ROS) (Li et al. 2022; Parwez et al. 2022). This raises the question of whether stress itself might inhibit juice sac initiation and growth. Stress conditions are known to affect cell growth and development by modulating the cell cycle, cell expansion, and differentiation (Shafqat et al. 2021). Moreover, stress influences secondary metabolism, which can provide protection against oxidative stress (Nehela and Killiny 2020).

While the transcriptomic analysis was performed in endocarp cells, the metabolomic analysis, due to technical constraints, was performed in the entire ovary/fruitlet excluding the exocarp (peel), diaphragm, and seeds (Online Resource 1). Therefore, the question arises whether the observed metabolomic changes also occur in the endocarp cells or whether the endocarp is characterized by distinct metabolite differences between Calabria and Yemenite citron across the analysed developmental stages. A partial answer to this question is provided by the joint pathway analysis, which integrates transcriptomic and metabolomic data and compares changes in metabolite levels with the expression of their corresponding biosynthetic genes (Katam et al. 2022). Metabolomic analysis of entire ovaries and fruitlets showed that TCA cycle metabolites were generally higher in *Yemenite* citron, whereas most major amino acids were more abundant in *Calabria*, except for aspartate and glutamate, which were higher in *Yemenite*. Furthermore, transcriptomic data showed that *Yemenite* endocarpal cells were enriched in processes related to photosynthesis, redox regulation, signalling, transport, and carbohydrate metabolism in at least three developmental stages. The joint pathway analysis,, confirmed that TCA cycle and energy metabolism pathways were enriched in *Yemenite* citron, suggesting that for these processes, endocarpal cells and entire ovaries/fruitlets showed similar metabolic behaviour. However, for amino acid metabolism, this correspondence was evident only for cysteine, methionine, tyrosine, and lysine; other amino acid pathways showed enrichment either in *Yemenite* or in both cultivars.

The TCA cycle is a central pathway in cellular respiration, converting pyruvate from carbohydrate breakdown into ATP (Salazar-Roa and Malumbres 2017). It also provides carbon and biosynthetic intermediates necessary for cell growth and division (Zhang and Fernie 2018). In addition, the TCA cycle plays a key role in responses to biotic and abiotic stresses, and its reprogramming is central in mitigating stress, including oxidative stress, through ROS scavenging (Zhang and Fernie 2023; MacLean et al. 2023). Moreover, galactose metabolism, which has been implicated in defence responses against stress, was enriched in *Yemenite* citron during anthesis according to the joint pathway analysis (Araniti et al. 2018; Zhang et al. 2015). While galactose itself was not identified in the metabolomic analysis, two related compounds—galactarate (mucic acid) and glucarate (saccharic acid)—were detected. Both compounds showed elevated levels in *Yemenite* ovaries and fruitlets compared to *Calabria* (Fig. S3). These sugar acids, which arise from the oxidation of glucose and galactose, are metabolically interconverted by galactarate dehydratase and glucarate dehydratase. Both compounds, or at least one of them, have been reported to accumulate under stress conditions, including oxidative stress (Araniti et al. 2018; Mellidou et al. 2021; Yadav et al. 2021; Rangani et al. 2020; Barros et al. 2023).

Additionally, several citrus orthologs of *Arabidopsis* stress-related genes were detected, including BETA-AMYLASE-like genes involved in stress-induced starch metabolism (Berndsen et al. 2025; David et al. 2022) and raffinose metabolism-related genes linked to various stress responses (Yan et al. 2022), further supporting the possibility of induced stress signalling in *Yemenite* citron.

## Candidate driver gene that might regulate JSs initiation

The above data, together with our previous publication, suggest that ABA and stress-like conditions may contribute to the suppression of juice sac initiation in *Yemenite* citron. However, it is also reasonable to assume that a regulatory mechanism promoting juice sac initiation exists in *Calabria* citron but not in *Yemenite*. This assumption formed the basis for focusing on TFs that were induced in the endocarp of *Calabria* compared to *Yemenite*. WGCNA identified 21 TFs in Group 4, enriched in *Calabria*, which could serve as driver genes for juice sac initiation and formation. Most of these TFs are involved in tissue development and growth, particularly in reproductive organs.

Several identified genes have been reported to regulate floral meristem formation in *Arabidopsis* and other plants. These include REM1, which functions in flower development (Mantegazza et al. 2014); VRN1/REM39, a DNA-binding protein involved in floral transition and later stages of flower development (Mantegazza et al. 2014); and LMI2, which promotes meristem identity transition from vegetative growth to flowering (Pastore et al. 2011). Additional TFs included NFL, a bHLH TF that controls flowering time via gibberellin signaling (Sharma et al. 2016), and FIL, which regulates meristem structural organization (Chen et al. 1999).

Other genes in this group are involved in cell development, specification, and proliferation. These include RGL1, a DELLA subfamily GRAS protein that restricts cell proliferation and expansion (Wen and Chang 2002); PDF2, expressed in the L1 layer of vegetative, floral, and inflorescence meristems, with a role in epidermal cell differentiation (Nagata and Abe 2023; Rombolá-Caldentey et al. 2014); WOX1, which regulates marginal meristem development (Dolzblasz et al. 2016; Wang et al. 2020); and PHB, which controls root and leaf tissue formation (Bertolotti et al. 2021; Hur et al. 2015).

Although juice sac primordia were first detected one week after anthesis, the regulatory mechanisms controlling this process are likely active at earlier stages of flower and ovary development, possibly even during the meristem transition stage. Therefore, genes functioning from meristem initiation through floral organ development and fruit set could be involved in regulating juice sac initiation.

Four TFs from Group 4 were selected for further analysis because, unlike most members of their gene families—which showed induction in *Yemenite* citron—these TFs were induced in three or four developmental stages in *Calabria* citron. TF2 and TF3 showed similar expression levels in whole ovaries and fruitlets of both cultivars but exhibited pronounced differences in endocarp tissue, suggesting specific roles in endocarpal cells. TF4 showed very low abundance in the ovaries of both cultivars, further supporting its endocarp-specific function, similar to TF2 and TF3.

These three TFs are associated with developmental processes. TF2, a BOLITA-like gene, encodes an ethylene-responsive transcription factor (ESR2) that may also regulate the cell cycle. TF3, an LMI1-like gene, encodes a putative homeobox-leucine zipper protein that regulates meristem identity and acts as a transcription factor in various biological processes (Xu et al. 2010). TF4is a NAC-domain transcription factor. Members of the family control multiple developmental processes, including meristem initiation and formation, ovule number, and carpel development (Cucinotta et al. 2018; Kamiuchi et al. 2014; Hibara et al. 2006; Raman et al. 2008). The gene shows high homology level to *CUC2* gene from *Arabidopsis* and *GOBLET* from tomato. These genes regulate secondary structure development in leaves, *CUC2* leaf serration, and *GOBLET*, leaflet formation in compound leaf, by controlling the formation boundary zones between secondary organs (Bilsborough et al. 2011; Ben-Gera et al. 2012). Interestingly, ectopic expression of *GOBLET* has been reported to generate a multi-carpel phenotype (Berger et al. 2009). Tomato and citrus, like most true-type fruits, are composed of carpels, which ontogenetically originate from leaves or share a common ontogenetic origin with leaves (Fahn 1990). Therefore, secondary structures on leaves and carpels may share a similar origin and/or be controlled by similar developmental mechanisms. A worth of investigation hypothesis is that TF4 is recruited in citrus for the initiation of juice sac primordia.

In contrast to TF2, TF3, and TF4, TF1 showed low expression even in the entire ovary of *Yemenite* citron, suggesting that its differential expression between cultivars may not be specific to the endocarp. Nevertheless, its involvement in juice sac initiation cannot be excluded, as its *Arabidopsis* ortholog (AT1G68640, PAN) is known to regulate floral organ development (Das et al. 2009; Maier et al. 2009; Chuang et al. 1999).

## Concluding remarks

The juice sac is a unique structure found exclusively in citrus fruits and absent from other fruit types. The regulation of juice sac initiation and development remains unclear and presents a challenge, given the complexity of the system and the limited availability of advanced experimental tools for studying this process. Nevertheless, the *Yemenite* citron, which does not develop juice sacs, provides a useful system for investigating juice sac initiation through physiological and biochemical approaches.

While the transcriptomic analysis in this study focused specifically on endocarp cells, the source tissue for juice sacs, the metabolomic analysis was performed on entire ovaries and fruitlets. Both the transcriptomic analysis and the combined analysis of transcriptomic and metabolomic data suggest that juice sac formation in *Calabria* citron is driven by growth-related processes such as cell division and proliferation. In contrast, *Yemenite* citron was characterized by the induction of stress-response mechanisms, which may be linked to elevated ABA levels, as shown in our previous publication (Assili et al. 2024).

This study also identified key transcription factors potentially involved in endocarp cell differentiation and juice sac initiation, including members of the GRAS, ERF, HD-ZIP, and NAC transcription factor families. These findings provide a foundation for future research into the molecular mechanisms controlling juice sac initiation in citrus fruits. For example, the possible involvement of specific TFs in juice sac initiation is currently being investigated using transgenic approaches. Additionally, broader comparisons involving more citron cultivars—both those that develop juice sacs and those that do not—may further improve our understanding of this mechanism.

## Supplementary Information

Below is the link to the electronic supplementary material.

Supplementary file1 (PDF 220 KB)

Supplementary file2 (XLSX 11 KB)

Supplementary file3 (XLSX 10641 KB)

Supplementary file4 (PDF 173 KB)

Supplementary file5 (XLSX 216 KB)

Supplementary file6 (XLSX 46 KB)

Supplementary file7 (XLSX 37 KB)

Supplementary file8 (XLSX 25 KB)

Supplementary file9 (XLSX 135 KB)

Supplementary file10 (XLSX 24 KB)

Supplementary file11 (XLSX 28 KB)

Supplementary file12 (PDF 386 KB)

Supplementary file13 (XLSX 71 KB)

Supplementary file14 (XLSX 43 KB)

Supplementary file15 (PDF 134 KB)

Supplementary file16 (PDF 178 KB)

Supplementary file17 (PDF 196 KB)

## Data Availability

Full results of the metabolomic analysis and the transcriptomic analyses, including accession numbers, *Arabidopsis* homologues, identity values, and fold-change values, are provided as Supplementary data. Additional information will be provided upon request.
