# Supplementary material for: Transcriptomic and metabolomic changes associated with the induction and initiation of juice sacs in citrus fruit
Source: Planta. 2026 May 5;263(6):149. doi: 10.1007/s00425-026-05008-9 (PMC13144201; doi:10.1007/s00425-026-05008-9)
Supplement: Supplementary file 3 — Supplementary file3 (XLSX 10641 KB) [file 425_2026_5008_MOESM3_ESM.pdf]

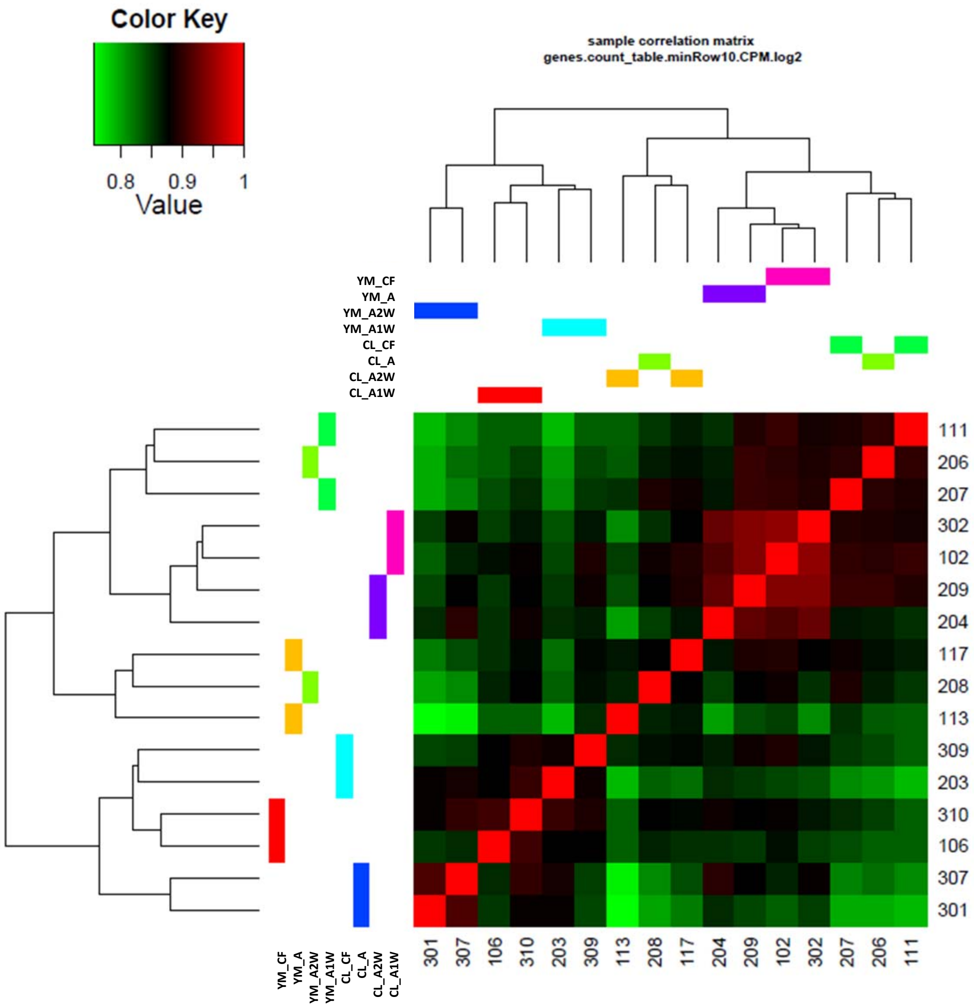

Online Resource 4. Transcriptomic analysis. Correlation matrix of the whole-transcriptome dataset obtained from two citron cultivars, Calabria (CL) and Yemenite (YM), sampled at four developmental stages: closed flowers (CF), anthesis (A), one week after anthesis (A1W), and two weeks after anthesis (A2W). The analysis compares expression values of the entire transcriptome (29,656 unigenes) across 16 samples, including two biological replicates per condition. Hierarchical clustering and the color scale represent the degree of correlation between samples.
