# Supplementary material for: Transcriptomic and metabolomic changes associated with the induction and initiation of juice sacs in citrus fruit
Source: Planta. 2026 May 5;263(6):149. doi: 10.1007/s00425-026-05008-9 (PMC13144201; doi:10.1007/s00425-026-05008-9)
Supplement: Supplementary file 11 — Supplementary file11 (XLSX 28 KB) [file 425_2026_5008_MOESM11_ESM.pdf]

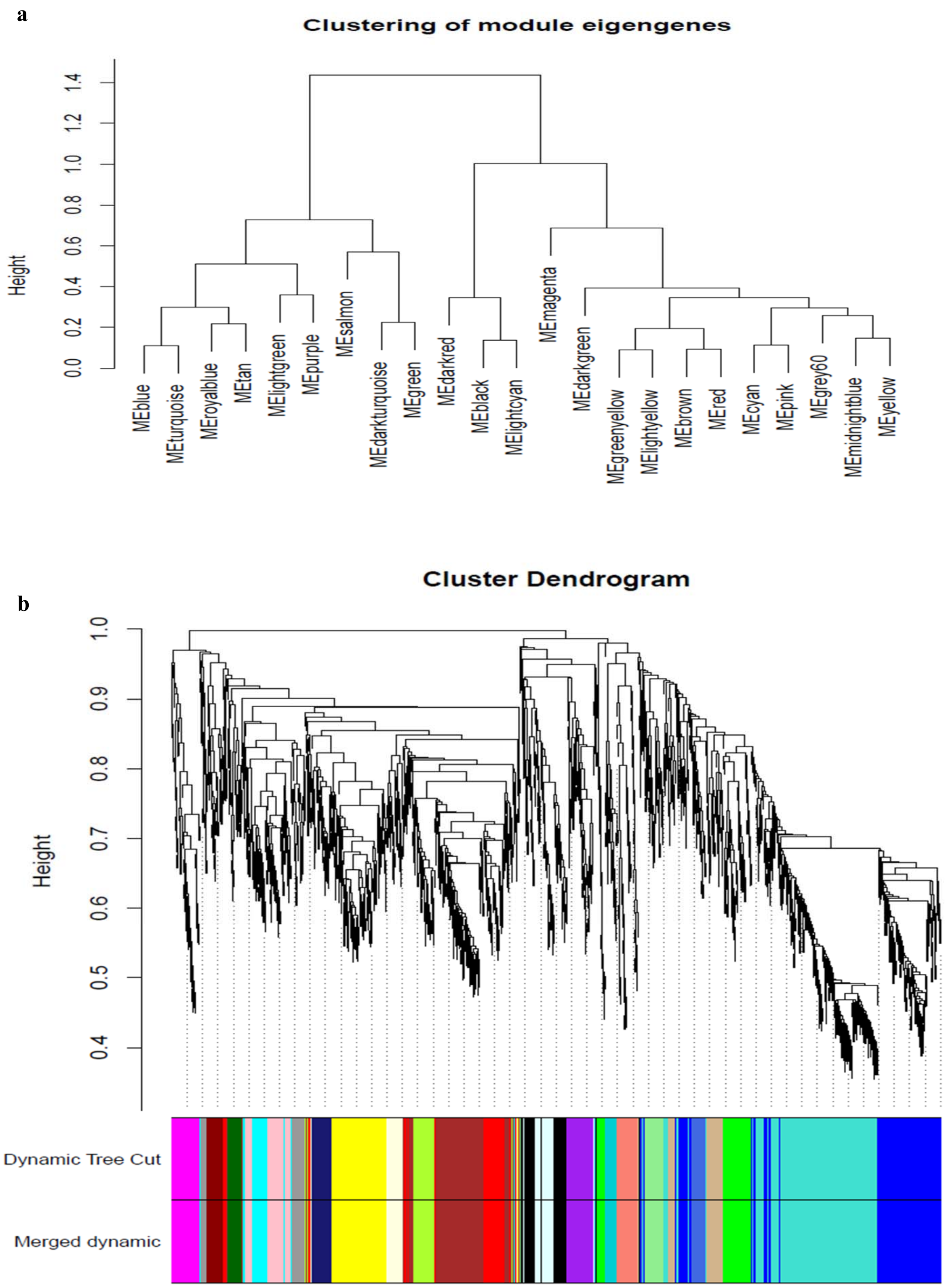

Online Resource 12. Weighted Gene Co-Expression Network Analysis (WGCNA) and module detection. (a) Hierarchical clustering of 23 module eigengenes (MEs). The y-axis represents the distance (1–TOM), and the x-axis indicates the different module eigengenes labeled according to module color (prefixed with ME). (b) Gene clustering and module assignment based on the topological overlap dissimilarity measure (1–TOM). The dendrogram branches represent groups of highly correlated or interconnected genes. The horizontal color bars indicate module assignment before and after merging. Grey modules represent genes that were not assigned to any module.
