# Supplementary material for: Transcriptomic and metabolomic changes associated with the induction and initiation of juice sacs in citrus fruit
Source: Planta. 2026 May 5;263(6):149. doi: 10.1007/s00425-026-05008-9 (PMC13144201; doi:10.1007/s00425-026-05008-9)
Supplement: Supplementary file 14 — Supplementary file14 (XLSX 43 KB) [file 425_2026_5008_MOESM14_ESM.pdf]

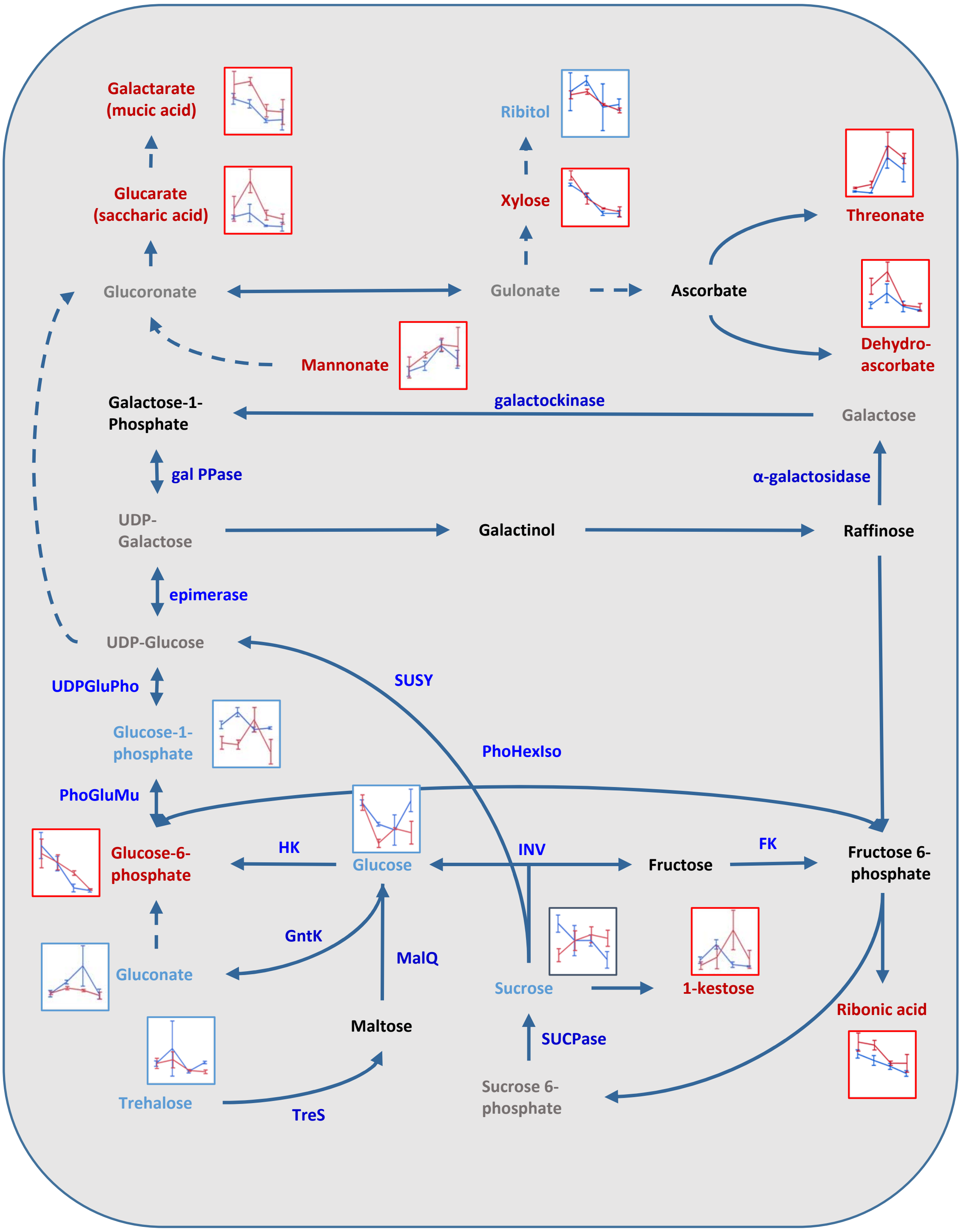

Online Resource 15. Schematic representation of sugar metabolism and related metabolites detected in citron tissues. Metabolites with higher overall abundance in Yemenite citron are indicated in red, those with higher abundance in Calabria citron in blue, and metabolites showing no change between cultivars in gray. For each metabolite, side charts display the mean values ± standard error across developmental stages, including closed flowers, anthesis, one week after anthesis, and two weeks after anthesis for both Calabria and Yemenite citrons.
