# Supplementary material for: Transcriptomic and metabolomic changes associated with the induction and initiation of juice sacs in citrus fruit
Source: Planta. 2026 May 5;263(6):149. doi: 10.1007/s00425-026-05008-9 (PMC13144201; doi:10.1007/s00425-026-05008-9)
Supplement: Supplementary file 16 — Supplementary file16 (PDF 178 KB) [file 425_2026_5008_MOESM16_ESM.pdf]

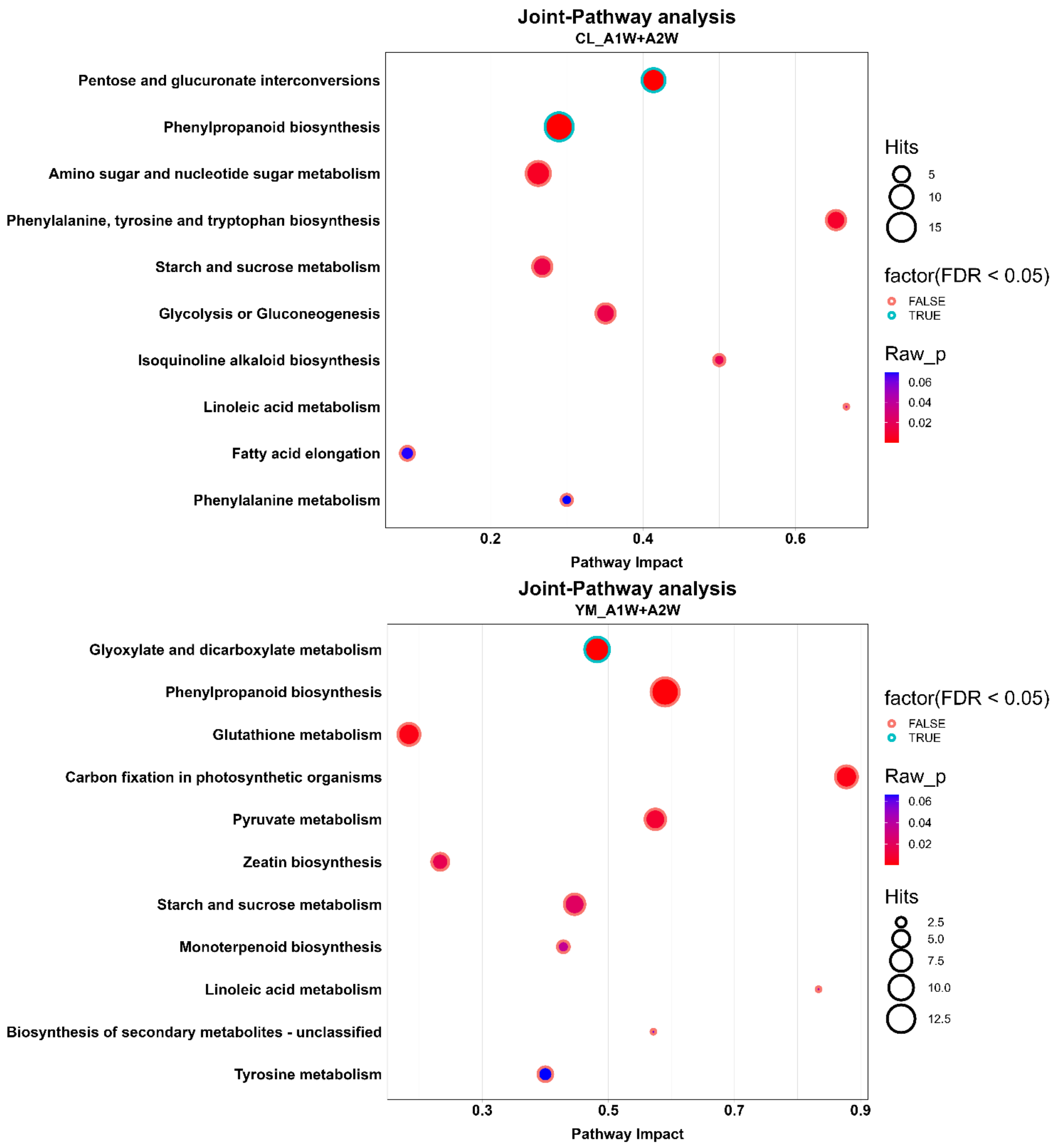

Online Resource 17. Integrated joint pathway enrichment analysis of transcriptomic and metabolomic data at one and two weekd post anthesis (A1W+A2W) in Calabria and Yemenite citron. Advanced bubble charts displaying significantly enriched metabolic pathways identified by joint pathway analysis of differentially expressed genes (DEGs) and differentially accumulated metabolites at the anthesis stage (A), performed using MetaboAnalyst 5.0. Separate analyses are shown for Calabria (CL_A1W+A2W) and Yemenite (YM_ A1W+A2W) citron. The x-axis represents pathway impact, reflecting pathway topology analysis (relative importance of matched genes and metabolites within the pathway). The y-axis lists the enriched metabolic pathways. Bubble size corresponds to the number of matched entities (“Hits”), including both DEGs and metabolites mapped to each pathway. Bubble fill color indicates enrichment significance based on raw p-values (color gradient from higher to lower p-values), while the bubble border denotes statistical significance after false discovery rate (FDR) correction (FDR < 0.05).
