# Supplementary material for: Transcriptomic and metabolomic changes associated with the induction and initiation of juice sacs in citrus fruit
Source: Planta. 2026 May 5;263(6):149. doi: 10.1007/s00425-026-05008-9 (PMC13144201; doi:10.1007/s00425-026-05008-9)
Supplement: Supplementary file 17 — Supplementary file17 (PDF 196 KB) [file 425_2026_5008_MOESM17_ESM.pdf]

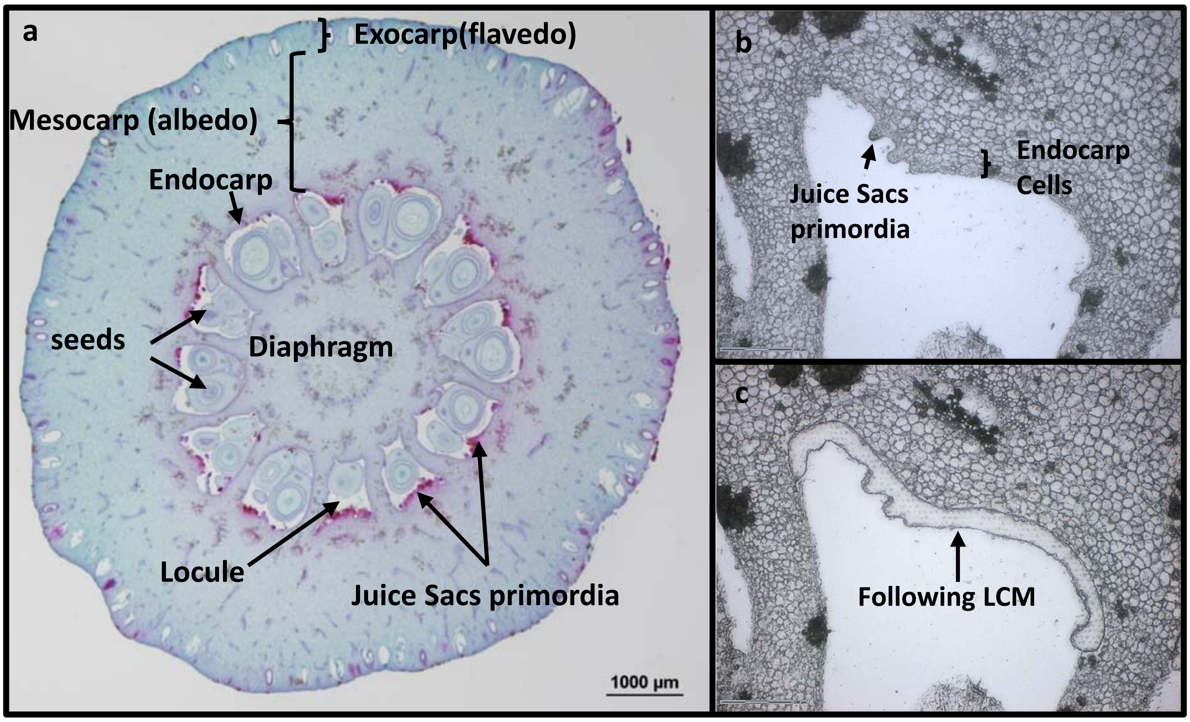

Online Resource 1. Structure of Calabria fruitlet. Microscopic cross-section of a Calabria citron fruitlet approximately three weeks post-anthesis showing the major fruit tissues (a). The exocarp (flavedo), mesocarp (albedo), endocarp, seeds, diaphragm, locule, and juice sac primordia are indicated. Enlarged cross-section of a single locule highlighting the endocarp cell layer and adjacent juice sac primordia prior to laser capture microdissection (LCM) (b). The same region following LCM is shown with a schematic indication of the dissected endocarp cells that were collected for transcriptomic analysis (c).
